# Single-Molecule Stoichiometry of Supramolecular Complexes

**DOI:** 10.1021/jacs.4c00611

**Published:** 2024-05-06

**Authors:** Alan McLean, Renata L. Sala, Brooke W. Longbottom, Alexander R. Carr, Jade A. McCune, Steven F. Lee, Oren A. Scherman

**Affiliations:** † Melville Laboratory for Polymer Synthesis, Yusuf Hamied Department of Chemistry, University of Cambridge, Lensfield Road, Cambridge CB2 1EW, United Kingdom; ‡ Yusuf Hamied Department of Chemistry, University of Cambridge, Lensfield Road, Cambridge CB2 1EW, United Kingdom

## Abstract

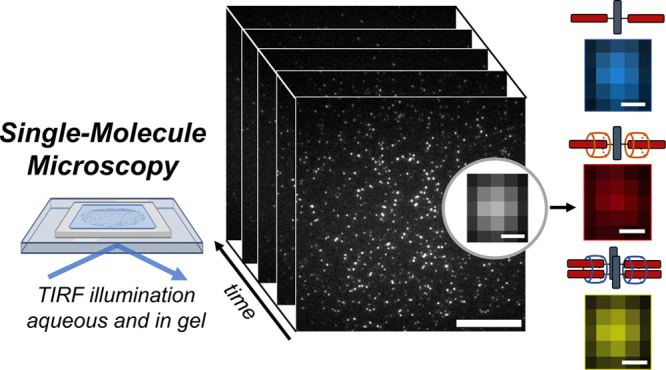

The use of single-molecule microscopy is introduced as a method to quantify the photophysical properties of supramolecular complexes rapidly at ultra low concentrations (<1 nM), previously inaccessible. Using a model supramolecular system based on the host–guest complexation of cucurbit[*n*]uril (CB[*n*]) macrocycles together with a fluorescent guest (**Ant910Me**), we probe fluorescent CB[*n*] host–guest complexes in the single molecule regime. We show quantification and differentiation of host–guest photophysics and stoichiometries, both in aqueous media and noninvasively in hydrogel, by thresholding detected photons. This methodology has wide reaching implications in aiding the design of next-generation materials with programmed and controlled properties.

Cucurbit[*n*]urils (CB[*n*]s) are macrocyclic host molecules formed from *n* glycoluril units joined through methylene bridges.^1−5^ CB[*n*]s have attracted much attention on account of their high binding affinities (up to 10^17^ M^–1^),^5−39^ wide range of guest molecules, and chemically robust nature.^4,5^ CB[7] and CB[8] homologues have shown particular promise on account of their ability to encapsulate larger guest molecules within their cavity.^4,5^ CB[7] can typically encapsulate one guest within its hydrophobic cavity, while the larger CB[8] is able to encapsulate two guest molecules simultaneously as shown in Figure 1A.^7,8^ The formation of CB[8]-homo- or heteroguest complexes is often exploited to unite chemical entities in a dynamic and controlled manner.^4,9−11^

**Figure 1:**
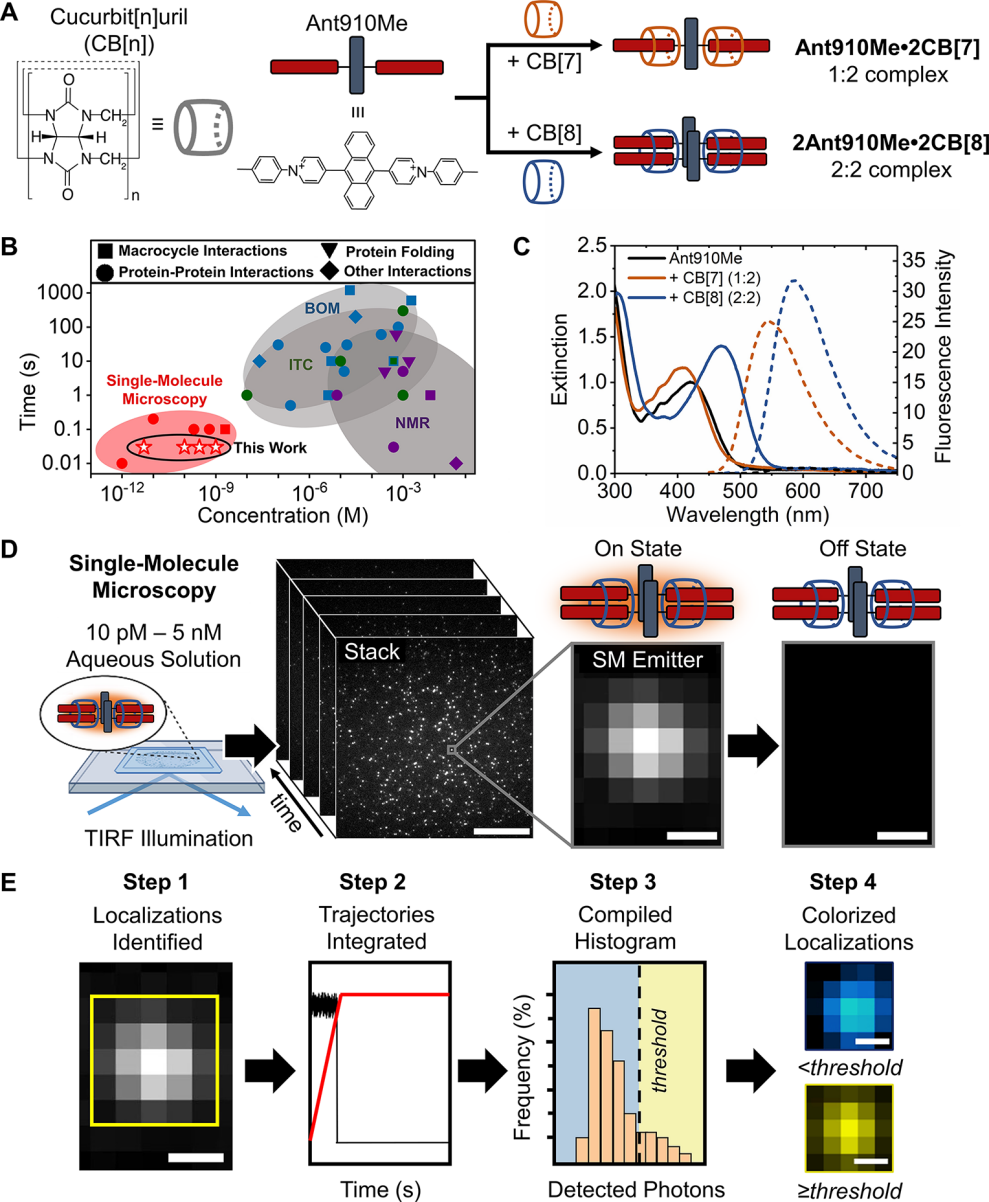
**A.** Schematic and chemical structures of cucurbit[*n*]uril (CB[*n*]) where *n* = 7 and 8 and the **Ant910Me** guest molecule (2 Cl^–^ counterions not shown). Host–guest binding occurs between **Ant910Me** and CB[7] (1:2) and CB[8] (2:2). **B.** Time resolution and concentration limits for various techniques used to study noncovalent interactions, including single-molecule microscopy (SMM), bulk optical measurements (BOM), isothermal titration calorimetry (ITC), and NMR spectroscopy. **C.** Absorption/emission from free **Ant910Me** and host–guest complexes of **Ant910Me** with CB[7] and CB[8]. **D.** Overview of SMM acquisition, where each molecule emits fluorescence (“on state”) until deactivated (“off state”), scale bar is 5 μm (stack) and 2 pixels in length (174 nm, localization) **E.** SMM processing pipeline, scale bars equal 2 pixels (174 nm).

Probing noncovalent interactions such as the host–guest chemistry of CB[*n*] has been largely limited to bulk ensemble measurements, Figure 1B. Typical tools used to study these interactions include 1D and 2D nuclear magnetic resonance spectroscopy (NMR), isothermal titration calorimetry (ITC) and bulk optical measurements (BOM) including UV/Vis and fluorescence spectroscopies, Figure 1B. These tools have limited capability to probe highly dynamic host–guest interactions, especially at lower concentrations <100 nM - 1 μM, Figure 1B. Quantification of host–guest interactions in relevant materials concentration regimes is critical to understand complex systems such as supramolecular polymer networks,^12−14^ supramolecular prodrugs,^15^ as well as colloidal hybrid interfaces^10,16^ and move toward the design of self-reporting and autonomous materials.^17^

One technique that enables facile, rapid quantification at ultra low concentrations (<nM) is single-molecule microscopy (SMM), Figure 1B. Compared to bulk spectroscopic or calorimetric measurements, SMM enables measurements that record the temporal dynamics of individual fluorescent molecules.^18−20^ With rapid acquisition times, a noninvasive nature, and an ability to image in solvated environments, SMM allows for visualization and measurement of native processes that would otherwise be unobservable. SMM has been widely employed in the biological domain to quantify biolabeled fluorescence,^21,22^ substrate–protein binding,^23^ DNA interactions,^21,22^ and protein counting.^24^ Determining the stoichiometry of proteins and their complexes by measuring stepwise photobleaching events^23^ has presented challenges to the field on account of the interference of high noise levels and photoblinking events, which impact reliability of the results. Researchers have addressed these issues to determine stoichiometry in protein complexes through various means including use of fluorescent proteins^25^ or integrating neural networks to deconvolute the data.^26^

There has been a recent drive to introduce SMM for imaging of materials on account of its noninvasive nature and ability to probe molecular-level interactions,^27,28^ however, the field is very much in its infancy. To date, there are only a few reports that have used SMM within materials e.g. to obtain high resolution images of block copolymers^27^ and thin film microdomains.^28^ However, the use of SMM as a quantitative tool to probe discrete host–guest complexes and their stoichiometry within materials has yet to be realized. SMM has the sensitivity to detect optically active host–guest complexes in low concentration regimes (1 pM - 10 nM) in time resolutions close to that of the dynamics of host–guest binding itself (ms time scales), Figure 1B. Using SMM could thus unveil information about host–guest complexation dynamics in real-time compared to bulk ensemble measurements. However, this requires the development of an alternative approach to determine guest stoichiometry than the measurement of stepwise photobleaching as multiple guests within a host–guest complex can become electronically coupled.

## Results and Discussion

Here we utilize SMM to visualize optically active CB[*n*] host–guest complexes. To achieve this, we first considered how we could access CB[*n*] host–guest complexes with controllable stoichiometries and optical properties that would be suitable for SMM, including absorptivity in the visible regime (λ_*max*_ > 400 nm) and high emission brightness (ϵ(λ_abs_)× ϕ_F_). CB[*n*] clamping of fluorescent guest molecules has been shown to greatly improve the quantum yield (ϕ_F_) and photostability of guests, while simultaneously promoting spectral tunability through molecular modifications.^9,29−35^ Since the introduction of the CB[8] 2:2 binding motif in 2017,^9^ we have demonstrated the ability to clamp a library of optically active guests together in a robust, predictable manner.^29−32^ We selected **Ant910Me** as a model optically active CB[*n*] guest molecule on account of its established 1:2 and 2:2 binding to CB[7] and CB[8] respectively,^30^ in addition to its high absorption cross-section and ultra high quantum yield (>90%) when excited in the blue, Figure 1C. We used SMM to obtain time-dependent fluorescent single-molecule timelapses of **Ant910Me·CB [***n***]** host–guest complexes both in solution and within a hydrogel material.

To be able to extract quantitative information from these timelapses, a stepwise imaging and analysis pipeline had to be developed, Figure 1D-E, Supporting Information Section 3. In brief, solutions were prepared at concentrations in which isolated emitters could be imaged until photobleached (<1 nM) using total internal reflection fluorescence (TIRF) excitation (30 Hz framerate; 45 s acquisition time), Figure 1D. A time-lapsed ‘stack’ of isolated emitters was obtained, Figure 1D. From this stack, the localizations were identified, Figure 1E Step 1, and their trajectories (signal/time) integrated to determine the total number of photons detected, Figure 1E Step 2. The photons detected across all localizations were compiled in a histogram and thresholded, Figure 1E Step 3. Finally, to enable easy visualization between samples, localizations had a threshold-discriminated two-color lookup table (LUT) applied based on the number of detected photons at the 90th percentile from the reference sample histogram, Figure 1E Step 4. This percentile was selected to minimize false positives, and can be evaluated using a quantitative approach, Supporting Information Section 2.8. We assigned the localizations below the threshold as blue for **Ant910Me** and red for **Ant910Me·2CB[7]**. Those above the threshold corresponded to **2Ant910·2CB[8]** which were assigned as yellow.

**Figure 2:**
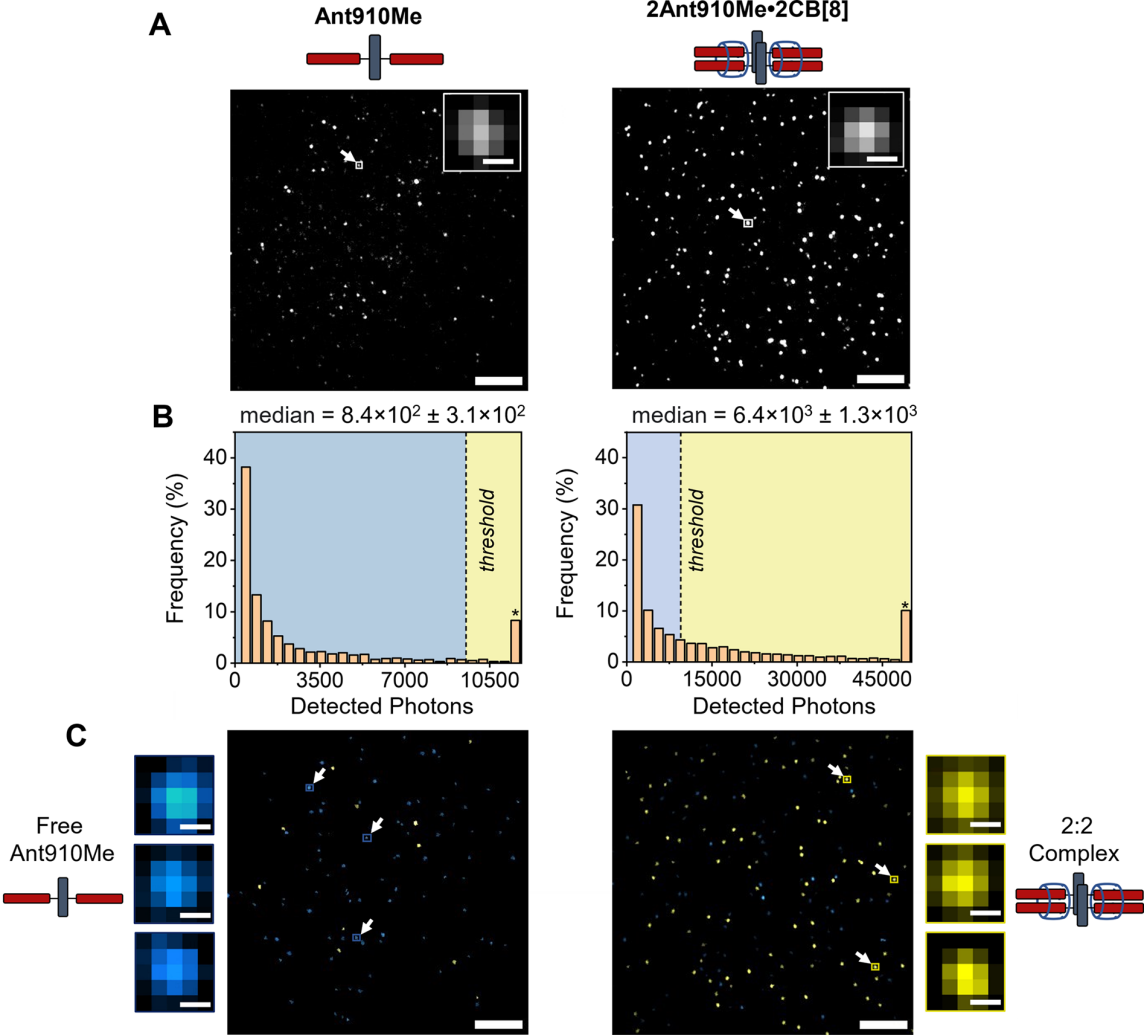
**A.** SMM localizations for **Ant910Me** and **2Ant910Me·2CB[8]** in aqueous solution at ultra low concentration (300 pM - 1 nM see Methods; scale bar is 5 μm and 2 pixels (174 nm) for inset images). **B.** Photon histograms where the *x*-axis is intensity (photons) and the *y*-axis is the frequency (in %) of the bin. The star (*) above the final bin indicates that the bin includes all values greater than. Median photons detected is provided in the upper right. The thresholded value (dashed line) was set to the 90th percentile of the **Ant910Me** free dye histogram (at 9526 detected photons). **C.** Thresholded localizations for **Ant910Me** and **2Ant910Me·2CB[8]** in aqueous solution, scale bar is 5 μm and 2 pixels (174 nm) for inset images.

Using SMM, we tested if we could differentiate **Ant910Me** as a free uncomplexed molecule and when complexed with CB[8] as a 2:2 complex. Evaluating single localizations, Figure 2A, single-step photobleaching trajectories were present, indicating the presence of single molecules or single complexes consistent with other SMM literature, Supporting Information Figure S9. Photon histograms compiled from hundreds of single molecules demonstrate that the **2Ant910Me·2CB[8]** complex detected more photons (6.4 × 10^3^ photons median) compared to free **Ant910Me** (8.4 × 10^2^ photons median), Figure 2B. Thresholding the **Ant910Me** histogram at its 90th percentile showed a clear difference in populations between **Ant910Me** and **2Ant910Me·2CB[8]**, Figure 2C. These data are consistent with the idea that thresholding the total integrated intensity allows for facile determination between molecules bound or unbound to its host complex. Differentiation between free **Ant910Me** and the **2Ant910Me·2CB[8]** complex using SMM can be attributed to differences on account of the host–guest binding stoichiometry with CB[8]. The 2:2 quaternary complex formed between **Ant910Me** and CB[8] results in two fluorophores (**Ant910Me**) per single-molecule localization compared to one fluorophore per localization for free **Ant910Me** in solution, Figure 2C (for intermediate stoichiometries see Supporting Information Section 2.6). Differentiation was similarly achieved between **Ant910Me·2CB[7]** and **2Ant910Me·2CB[8]** using the same methodology, Supporting Information Figure S16.

To further validate the scope of SMM as a tool to identify CB[*n*] host–guest stoichiometry, we introduced adamantylamine (**ADA**) (K_a_ > 10^12^ M^–1^),^36,37^ a nonfluorescent competitive guest, to disrupt the host–guest complexes, Supporting Information Figures S18, S19. The histogram and median detected photons showed a dramatic change close to the free **Ant910Me** baseline upon the addition of **ADA**, indicating that **Ant910Me** was displaced from the CB[8] cavity. Importantly, **ADA·CB[8]** produced no single-molecule localizations at relevant concentrations, validating that the change was solely attributed to a change in the **Ant910Me** environment (Supporting Information Figure S8).

**Figure 3:**
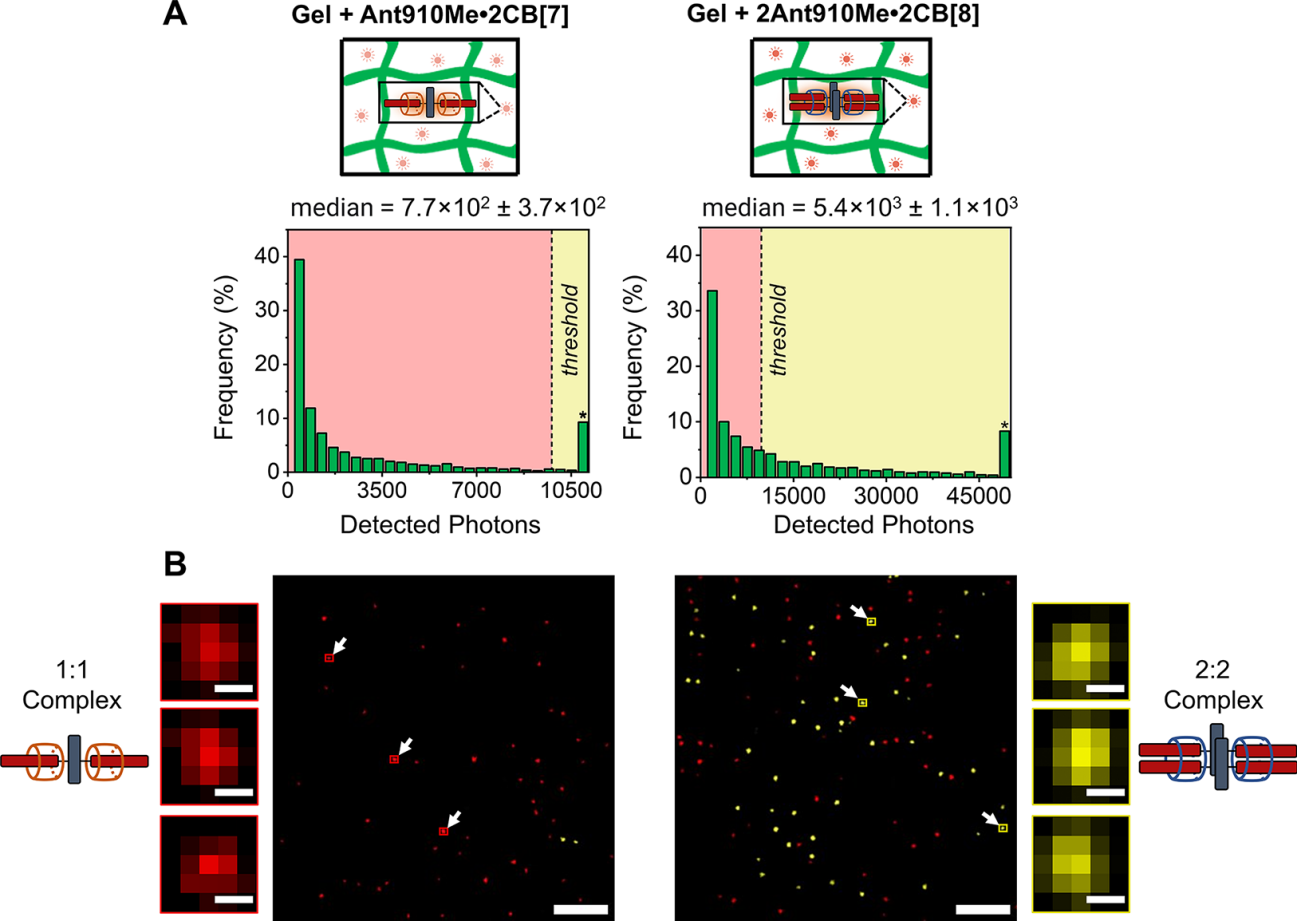
**A.** Depiction of **Ant910Me·2CB[7]**, and **2Ant910Me·2CB[8]** in agarose hydrogel and associated photon histograms in 3 wt% agarose solution. The thresholded value (dashed line) was set to the 90th percentile of the **Ant910Me·2CB[7]** agarose histogram at 9786 detected photons. **B.** Thresholded localizations for agarose samples (**Ant910Me·2CB[7]** and **2Ant910Me·2CB[8]** respectively), scale bar is 5 μm and 2 pixels (174 nm) for inset images.

Having proven the ability of SMM to differentiate CB[*n*] host–guest stoichiometry, we were keen to apply SMM to probe host–guest interactions within a material. To do so, we dispersed **Ant910Me·2CB[7]** and **2Ant910Me·2CB[8]** as free, discrete host–guest complexes within an agarose hydrogel. The SMM histograms and median detected photons from **Ant910Me·2CB[7]** and **2Ant910Me·2CB[8]** complexes remained similar when encapsulated within the agarose hydrogel compared to in aqueous solution, Figure 3A. As in aqueous solution, we were able to differentiate 1:2 and 2:2 CB[*n*] host–guest complexation stoichiometry within the hydrogel by thresholding the detected photons of each sample, Figure 3B. **Ant910Me·2CB[7]**, where only one fluorophore is present, is composed of predominantly red-generated localizations (LUTs), Figure 3B. In contrast, **2Ant910Me·2CB[8]** had more yellow-generated localizations (LUTs) above the threshold, Figure 3B. Our results demonstrate that the differentiation of optically active CB[*n*] host–guest stoichiometries through SMM can be achieved within a hydrogel medium at ultralow concentrations without photophysical perturbation. This is particularly significant as SMM could be used to probe dynamics in which macrocycles like CB[*n*] have been used as an active cross-linking component paired with guests at precise stoichiometric ratios such as in gels,^12,13,38^ drug delivery platforms,^10,15^ and colloidal interfaces.^16^ The ability to resolve the dynamics of noncovalent complexes in realtime should be of great significance in the development of self-reporting and autonomous materials.

## Conclusions

We report a robust, quantitative method for discriminating fluorescent host–guest CB[*n*] stoichiometries at ultra low concentrations (<1 nM) through single-molecule microscopy (SMM). Using SMM, we show clear differentiation between stoichiometries with a single fluorophore and two fluorophores, which reflect differences in the host–guest complexes formed. We demonstrate that our technique can not only be used to probe host–guest complexes in aqueous solution, but also host–guest complexes within a material. With facile sample preparation, noninvasive acquisition, and sensitive temporal resolution, single-molecule methodologies enable the study of host–guest systems at ultra low concentrations and have the potential to greatly further the understanding of host–guest chemistries in their native environment.

## Supporting Information Available

1: Materials and Methods, 2. Supporting Information: Figures and Tables 3. Appendix: Appendix I: FIJI Macro Script, Appendix II: Matlab Script, Appendix III: Getting Started: Beginner-Friendly Code Guide. (PDF)
