# Supplementary material for: Single-Molecule Stoichiometry of Supramolecular Complexes
Source: J Am Chem Soc. 2024 May 6;146(19):12877–82. doi: 10.1021/jacs.4c00611 (PMC11100007; doi:10.1021/jacs.4c00611)
Supplement: Supplementary file 1 — ja4c00611_si_001.pdf [file ja4c00611_si_001.pdf]

Supporting Information: Single-Molecule Stoichiometry of Supramolecular Complexes

Alan McLean,† Renata L. Sala,† Brooke W. Longbottom,† Alexander R. Carr,† Jade A. McCune,† Steven F. Lee,*,‡ and Oren A. Scherman*,†

†*Melville Laboratory for Polymer Synthesis, Yusuf Hamied Department of Chemistry, University of Cambridge, Lensfield Road, Cambridge CB2 1EW, United Kingdom*

‡*Yusuf Hamied Department of Chemistry, University of Cambridge, Lensfield Road, Cambridge CB2 1EW, United Kingdom*

E-mail: sl591@cam.ac.uk; oas23@cam.ac.uk

Contents

1 Materials and methods **S4**

1.1 Materials S4

1.2 Methods S5

Experimental Techniques S5

Nuclear Magnetic Resonance Spectroscopy (NMR) S5

UV/Vis and Fluorescence Spectroscopy S5

Fluorescence Lifetime Imaging Microscopy (FLIM) S5

Single-Molecule Microscopy S6

Sample Preparation S8

Synthesis of Ant910Me S8

UV/Vis and Fluorescence titration experiments S8

Sample preparation for Single-Molecule Microscopy S9

Preparation of agarose hydrogels S9

Single-Molecule Data Processing S10

2 Supporting Information Figures and Tables **S11**

2.1 Techniques to probe non-covalent interactions S11

2.2 UV/Vis and fluorescence titrations for Ant910Me with CB[7] and CB[8] . . . S12

2.3 FLIM measurements of free and complexed Ant910Me S14

2.4 Control experiments for single-molecule imaging S16

2.5 Example trajectories of 2Ant910Me·2CB[8] S17

2.6 SMM of Ant910Me and CB[8] at various stoichiometric ratios S18

2.7 SMM of 2Ant910Me•2CB[8] complexes at various concentrations S20

2.8 SMM Histogram Disjoints as a Function of Threshold (%) S21

2.9 SMM comparison of Ant910Me•2CB[7] and 2Ant910Me•2CB[8] S23

2.10 SMM Evaluation of Rho6G•CB[7] S24

2.11 Competitive displacement study S25
2.12 SMM comparison of free Ant910Me and 2Ant910Me•2CB[8] in Agarose . . . S27
2.13 Additional Single-Molecule Images S28
2.13.1 Single-Molecule Images for Figure 2 . . . . S28
2.13.2 Single-Molecule Images for Figure 3 . . . . S32

3 Appendix: Image Analysis Procedure S36

3.1 FIJI Macro Script S36
3.1.1 Intensity and Global Background Correction S36
3.1.2 Thresholding Prep: Noise Determination, Stack Processing S38
3.1.3 Localization Selection and Output S38
3.2 Matlab Script S39
3.2.1 Script 1: 2D Gaussian Fitting and Determining Median Detected Photons S40
3.3 Getting Started: Beginner-Friendly Code Guide S40
Part 1: FIJI S40
Part 2: Matlab S41

References S44

1 Materials and methods

1.1 Materials

Unless otherwise specified, all materials were purchased from commercial suppliers as detailed below and were used without further purification. 9,10-dibromoanthracene (Sigma-Aldrich, purity: 98%), 4-pyridine boronic acid (Sigma-Aldrich, purity: 90%), potassium carbonate (K_2CO_3) (anhydrous, Fisher, purity: >99%), tetrakis(triphenylphosphine)-palladium(0) ($Pd(PPh_3)_4$) (Alfa Aesar, purity: 99.8%), 1-chloro-2-4-dinitrobenzene (Acros Organics, purity, 99%), p-toluidine (Fluka Analytical, Spectrophotometric Grade, purity: >99.0%), 1-adamantylamine (ADA) hydrochloride (Sigma-Aldrich, purity >98%), sodium 3-(trimethyl silyl)propane-1-sulfonate (DSS, NMR Standard, purity: 98%), pyridine (Acros Organics, Extra Dry, purity: 99.5%), agarose (Sigma-Aldrich), rhodamine 6G (Lambda Physik), HPLC grade water (Fisher). Cucurbit[7]uril (CB[7]) and cucurbit[8]uril (CB[8]) were prepared and purified using a previously reported method.[S1,S2]

1.2 Methods

Experimental Techniques

Nuclear Magnetic Resonance Spectroscopy (NMR)

^{1}H NMR spectra were acquired in heavy water (D_2O) at 298 K and recorded on a Bruker AVANCE 500 with TCI Cryoprobe system (500 MHz) being controlled by TopSpin2.

UV/Vis and Fluorescence Spectroscopy

UV/Vis and fluorescence spectra were recorded on a Horiba Duetta fluorescence and absorption spectrometer using 1 cm pathlength quartz cuvettes (ThorLabs Model CV10Q35FE) at 298 K.

Fluorescence Lifetime Imaging Microscopy (FLIM)

Fluorescence lifetime imaging microscopy (FLIM) was performed on a PicoQuant MicroTime 200 time-resolved fluorescence microscope with an Olympus IX73 body and 60x water immersion objective (Olympus Model UPlanSApo; NA=1.20). For all samples, 488 nm picosecond pulsed excitation (5-40 MHz rep. rate) was used and a 512 nm longpass filter was used to collect emission on a PMA Hybrid-40 detector with a HydraHarp 400 TCSPC module. For imaging, samples were drop-deposited at 20 μM on a glass slide (VWR Cat No. 631-0122, 20 mm x 20 mm, Thickness No. 1) suspended in a slide holder. FLIM was performed in Z-scanning mode. This mode allows the user to scan from the surface of the glass slide (identified at the position of the surface Airy disk) into the bulk. Lifetime data was determined from fitting in the PicoQuant SymPhoTime 64 software and plots/fits were exported and post-processed (normalized, log-plot taken).

Single-Molecule Microscopy

Microscope Protocol Single-molecule microscopy was performed on a Nikon Eclipse Ti2 using a 100x TIRF objective (Nikon CFI Apochromat TIRF 100XC Oil; NA=1.49) equipped with a 4-line fiber optic laser source (405 nm; LuxXplus 405-120 diode laser, 488 nm; LuxXplus 488-200 diode laser, 561 nm; OBIS 561-150 DPSS laser, 640 nm; LuxXplus 640-140 diode laser, Omicron LightHUB-6), dichroic single pass filter (405 nm), dichroic quadpass filter (405, 488, 561, and 640 nm), longpass emission filters (440, 481, 561, 640 for each laser line, respectively), and a Photometrics Prime BSI sCMOS camera. A quarter waveplate was used to turn the linear polarization output to circularly polarized excitation (not depicted in Figure **S1**). For this study, samples were illuminated at 488 nm excitation in the TIRF plane using 30 ms exposure times over 1500 frames with 2 x 2 binning. Under these conditions, a single binned pixel corresponds to 87 nm. Power density was approximately 0.2 kW/cm^2. Glass slides (VWR Cat No. 631 − 0124, 22 mm x 22 mm, Thickness No. 1) were used for single-molecule experiments and plasma oxidized prior to sample handling from an air plasma source for >10 minutes for cleaning (Diener Atto, 200 W).

We note that the critical quantity in SMM is the total integrated photons on the detector. This is a complex combination of the intrinsic properties of the individual fluorophore, including the photon fluence extinction coefficient, fluorescent lifetime, and the fluorescence quantum yield. Typically, a value of around 300-500 detected photons above background per localization is reasonable to achieve accurate single molecule detection. In our study we find that we can detect an average of ca. 700-800 photons with free Ant910Me and Ant910Me•CB[7], both above the detection threshold and the number of photons detected for 2Ant910Me•2CB[8] was even greater at 6400.

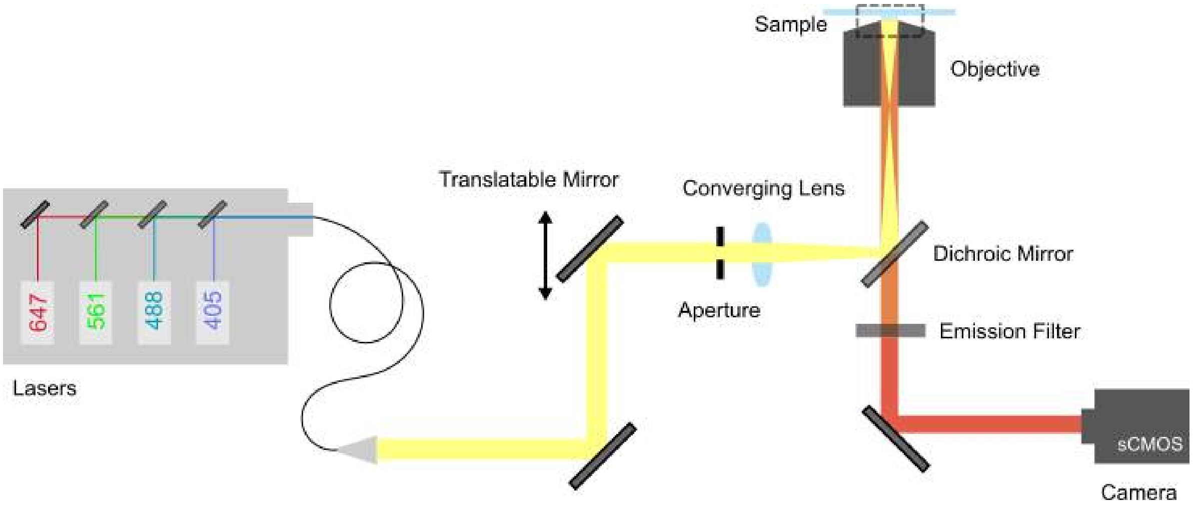

Figure S1: Schematic of single-molecule set-up used for quantitative single-molecule imaging.

Single-Molecule Set-Up for Agarose Hydrogel Imaging Figure **S2** depicts the steps for imaging of agarose hydrogels with post-loaded fluorophore (Ant910Me and Ant910Me•CB[n] complexes).

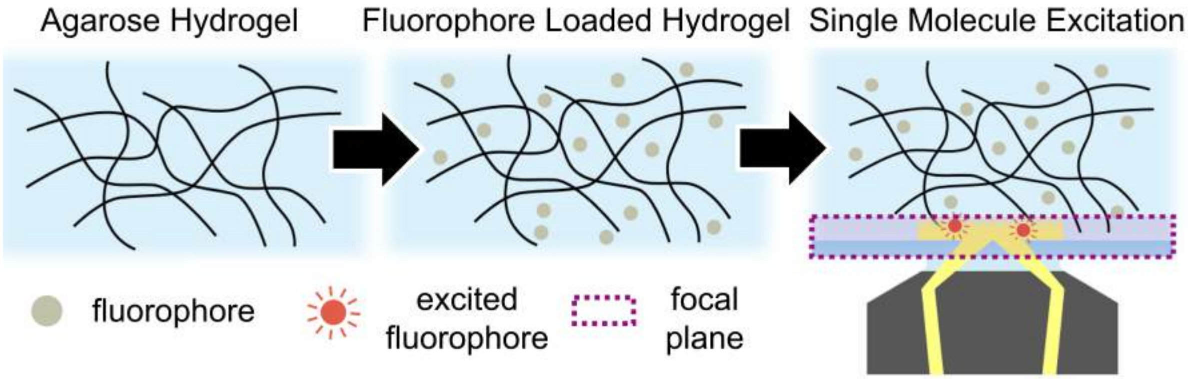

Figure S2: Schematic of single-molecule imaging setup for agarose hydrogels with fluorophore (Ant910Me, Ant910Me•2CB[7] or 2Ant910Me•2CB[8]) post-loaded.

Sample Preparation

Synthesis of Ant910Me

Ant910Me was synthesised following previously reported literature procedures published by our group.[S3–S5] As exemplified in Figure **S3**, the synthesis of Ant910Me is a three step-synthesis, starting with a Suzuki-Miyaura cross-coupling of two pyridin-4-yl groups onto the Ant910 fluorophore core, followed by the transformation of the pryidin-4-yl groups into arylpyridinium salts through a Zincke reaction. ^{1}H NMR matched previous literature reports: ^{1}H NMR (D_2O, 500MHz) 9.39 (d, J = 7.0 Hz, 4H), 8.46 (d, J = 6.9 Hz, 4H), 7.83 (d, J = 8.6 Hz, 4H), 7.79 - 7.73 (m, 4H), 7.70 – 7.61 (m, 8H), 2.55 (s, 6H).

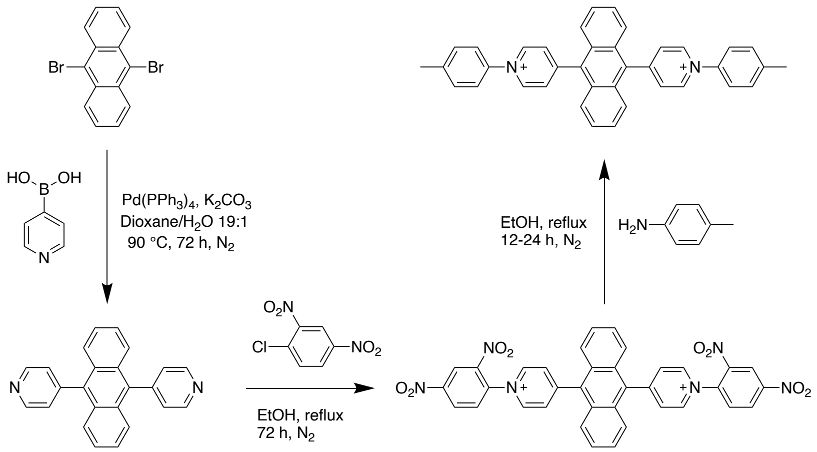

Figure S3: Synthetic route to Ant910Me based on previous literature reports.[S3–S5] Note Cl^- counterions have been omitted for clarity.

UV/Vis and Fluorescence titration experiments

The concentrations of both host (CB[n] (n=7,8)) and guest (Ant910Me, ADA) stock solutions were determined by ^{1}H NMR spectroscopy using commonly employed NMR standards (DSS, pyridine) prior to UV/Vis and fluorescence titrations. Fluorescence titration experiments were performed using a 5 μM solution of Ant910Me into which CB[n] (n=7,8) was titrated. For competitive guest displacement studies, adamantylamine (ADA) was added to a preformed sample of 2Ant910Me•2CB[8] (5 μM).

Sample preparation for Single-Molecule Microscopy

1 μM solutions of Ant910Me, Ant910Me•2CB[7] and 2Ant910Me•2CB[8] were prepared in HPLC grade water and diluted to pM/nM concentrations for measurements. 50 μL of each solution was drop-deposited on a plasma cleaned slide in the incubation gasket interior (Bio-Rad Frame-Seal Incubation Chambers, Cat No. SLF0201). Sample concentrations were optimized for appropriate single-molecule density and were as follows: Ant910Me : 1 nM, Ant910Me•2CB[7]: 600 pM, 2Ant910Me•2CB[8]: 300 pM.

For agarose experiments, the agarose gel was first formed before being injected with a 50 μL solution of either Ant910Me or Ant910Me•CB[n] complex. For Ant910Me and Ant910Me•2CB[7] 100 nM solutions were used, for 2Ant910Me•2CB[8] a 10 nM solution was used. For Rhodamine 6G experiments, a 1 nM solution was prepared and combined with CB[7] (2 nM) to promote complexation. Control experiments of the HPLC-grade water alone were run before every experiment to confirm no background fluorescence signal was coming from the solvent alone. Additional control experiments to measure CB[7] and CB[8] alone were performed to confirm no background fluorescence signal was coming from the macrocycles. This was done by depositing samples of each macrocycle at concentrations above the highest relevant single-molecule concentrations and measuring their single-molecule signal.

Preparation of agarose hydrogels

Agarose hydrogels were prepared at 3% w/v by dissolving low gelling temperature agarose powder in HPLC-grade water at 60 °C (e.g. 300 mg in 10 mL for 3%). 20-50 mL of hot agarose solution was pipetted into an incubation gasket (Bio-Rad Frame-Seal Incubation Chambers, Cat No. SLF0201) held on plasma-oxidized slides. The agarose hydrogel was incubated at 5 °C for 30 minutes to allow for the gel to set before being utilized for subsequent experiments. The agarose gel single-molecule background was tested before each experiment (before injecting the fluorescent dye/complex). Agarose gels displayed a level of single-molecule background signal, in some cases up to 10 - 20 localizations. The signal observed

was never greater than 10 % of the total localizations identified and was significantly below 10 % for the gels containing 2Ant910Me•2CB[8] complexes.

Single-Molecule Data Processing

Single-molecule data was processed using two independent scripts. The first script, a FIJI-compatible macro, was used to correct intensity and global background of the raw image, spatially identify the bright spots and their local background, and extract their single-molecule trajectories. Matlab scripts were used to determine the median detected photons and error. Sections in the Appendix describe the scripts in more detail.

2 Supporting Information Figures and Tables

2.1 Techniques to probe non-covalent interactions

Within Figure 1 in the main manuscript a summary of techniques use to probe non-covalent interactions is presented. Below is an expanded version of that figure with the corresponding references.

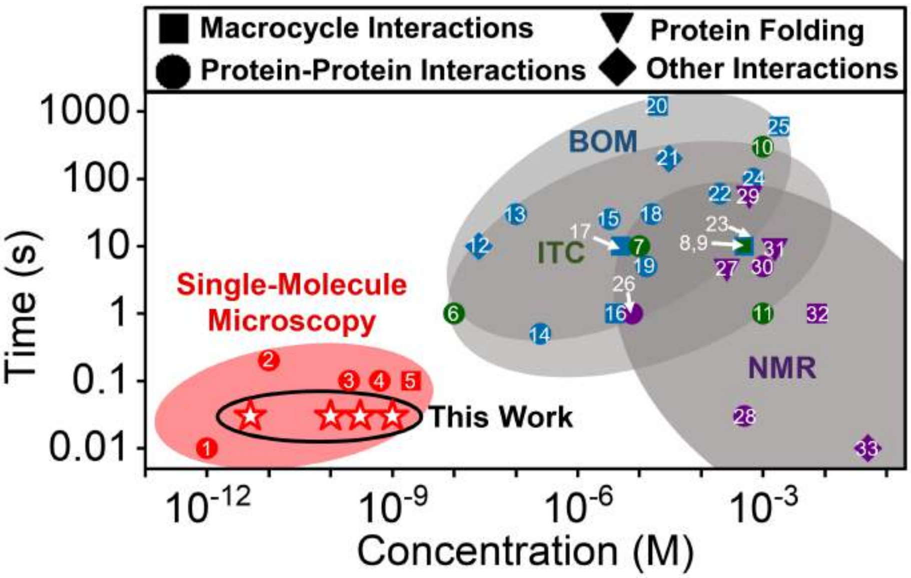

Figure S4: Expanded figure of techniques used to probe non-covalent interactions in real-time with references for each data point. Single-molecule microscopy (1-5),[S6–S10] isothermal titration calorimetry (ITC, 6-11),[S11–S16] bulk optical measurements (BOM) such as absorbance (UV/Vis) and fluorescence spectroscopy (12-25),[S17–S30] and nuclear magnetic resonance (NMR) spectroscopy (26-33).[S31–S38]

2.2 UV/Vis and fluorescence titrations for Ant910Me with CB[7] and CB[8]

As shown in Figure **S5** and Figure **S6**, titration of CB[7] (A & B) and CB[8] (C & D) into a solution of Ant910Me resulted in shifts of UV/Vis absorbance (Figure **S5**) and enhancement of fluorescence (Figure **S6**) indicating binding of the Ant910Me within the CB[n] (n=7,8) cavity. Plotting the change in absorption (Figure **S5** B & D) and fluorescence (Figure **S6** B & D) against the molar equivalents of CB[n] (n=7,8) shows a stoichiometric saturation in both cases. This occurs at a 1:2 ratio of Ant910Me:CB[7] (Figure **S5** B and Figure **S6** B) and at a 2:2 ratio of Ant910Me:CB[8] (Figure **S5** D and Figure **S6** D) indicating the formation of Ant910Me•2CB[7] and 2Ant910Me•2CB[8] complexes, respectively. These data are consistent with previous reports.[S4]

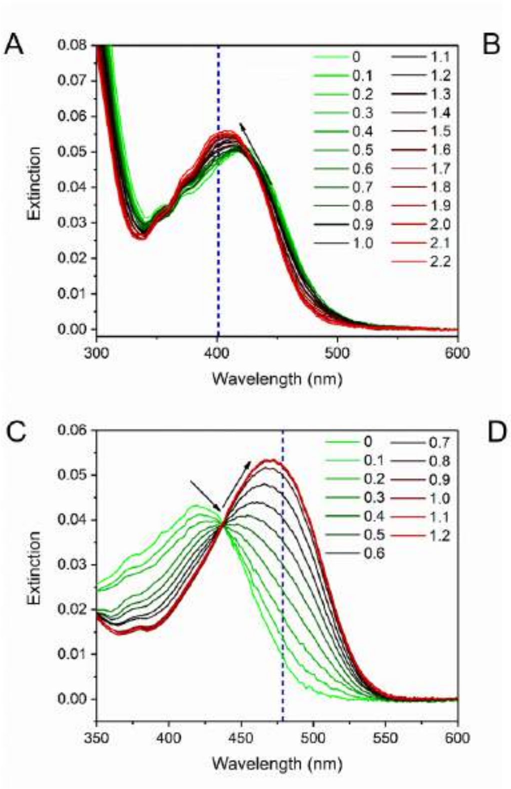

Figure S5: UV/Vis titration of CB[7] (**A** & **B**) and CB[8] (**C** & **D**) into Ant910Me. Full UV/Vis spectra are shown on the LHS (**A** & **C**), while on the RHS (**B** & **D**) the intensity is plotted at a chosen wavelength (denoted with a blue dashed line in plots **A** & **C**) against the molar ratio of CB[7] (**B**) and CB[8] (**D**).

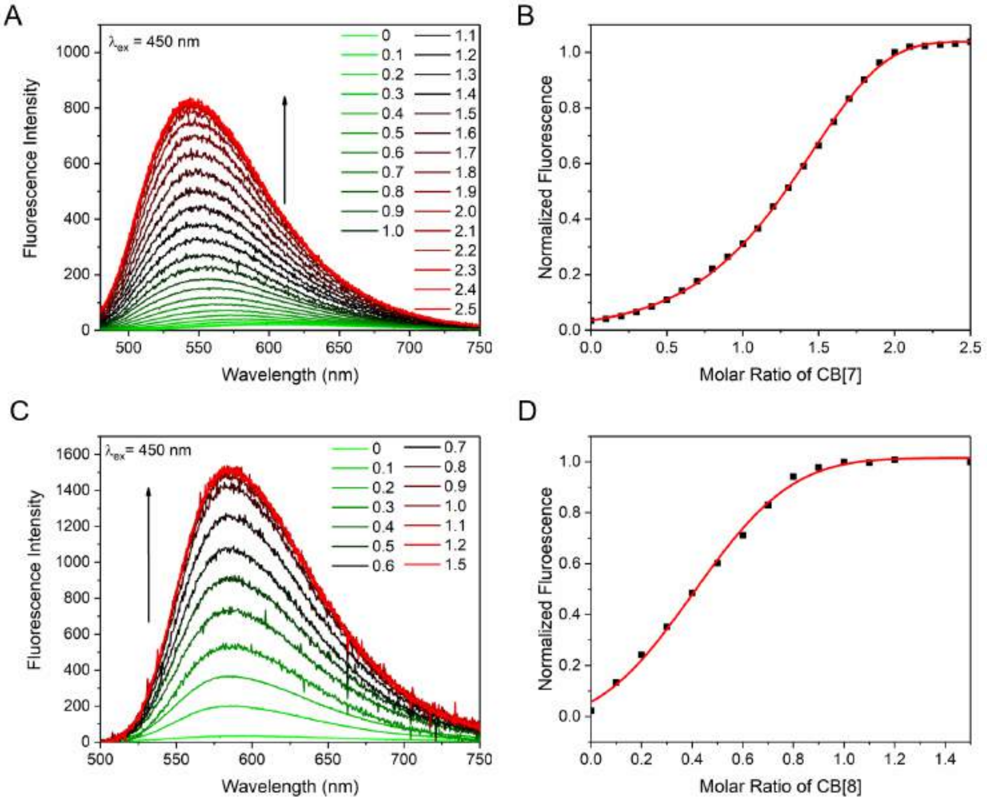

Figure S6: Fluorescence titration of CB[7] (**A** & **B**) and CB[8] (**C** & **D**) into Ant910Me. Full spectra are shown on the LHS (**A** & **C**), while on the RHS (**B** & **D**) the normalized fluorescence intensity is plotted against the molar ratio of CB[7] (**B**) and CB[8] (**D**).

2.3 FLIM measurements of free and complexed Ant910Me

FLIM has been established as a methodology to probe molecular adsorption to surfaces at interfaces, Figure **S7**A.[S39] Using FLIM, we measured the fluorescence lifetime of the Ant910Me free dye, Ant910Me•2CB[7] and 2Ant910Me•2CB[8] complexes at the glass surface and in bulk aqueous solution, Figure **S7** B & C. The results, summarized in Table S1, show that there are significant changes in the fluorescence lifetime of the Ant910Me free dye at the glass surface compared to in bulk aqueous solution. The lifetime of Ant910Me increases significantly on the glass surface on account of decreased intramolecular rotation from molecular adsorption to the glass surface, consistent with literature reports for other free dyes.[S39] In contrast, the lifetimes of Ant910Me•2CB[7] and 2Ant910Me•2CB[8] complexes remain consistent both at the glass surface and in bulk aqueous solution.

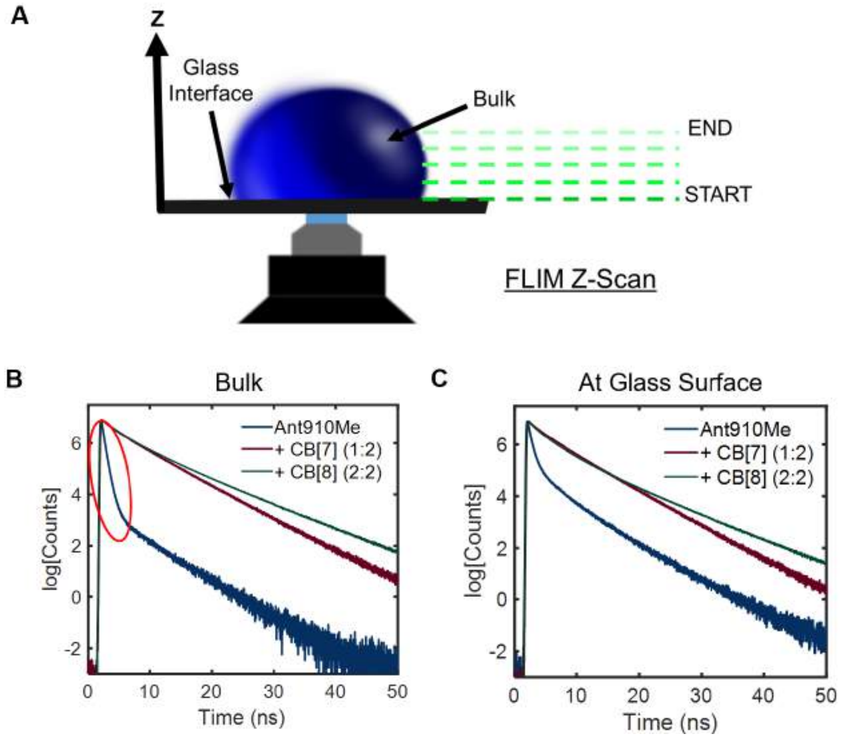

Figure S7: A Schematic depicting FLIM set-up used **B** Fluorescence lifetime plots of bulk measurements **C** Fluorescence lifetime plots of measurements at the glass surface

Table S1: Summary of fluorescence lifetimes of Ant910Me, Ant910Me•2CB[7] and 2Ant910Me•2CB[8] complexes in bulk aqueous solution and at the glass surface.
We note that CB[8] does exhibit three-component decay on the glass surface, but when the first two components are factored together by their relative intensities, they have a combined lifetime of 3.7 ns.

Sample	Fluorescence Lifetime (Bulk) ns (± 0.2 ns)	Fluorescence Lifetime (Glass Slide) ns (± 0.2 ns)
Ant910Me	0.6 ns (99%)	0.7 ns (61%)
	5.3 ns (1%)	3.6 ns (24%)
		7.8 ns (15%)
Ant910Me•2CB[7]	3.4 ns (29%)	3.0 ns (30%)
	8.0 ns (71%)	7.6 ns (70%)
2Ant910Me•2CB[8]	3.8 ns (46%)	1.5 ns (19%)
	10.5 ns (54%)	4.6 ns (46%)
		10.7 ns (36%)

2.4 Control experiments for single-molecule imaging

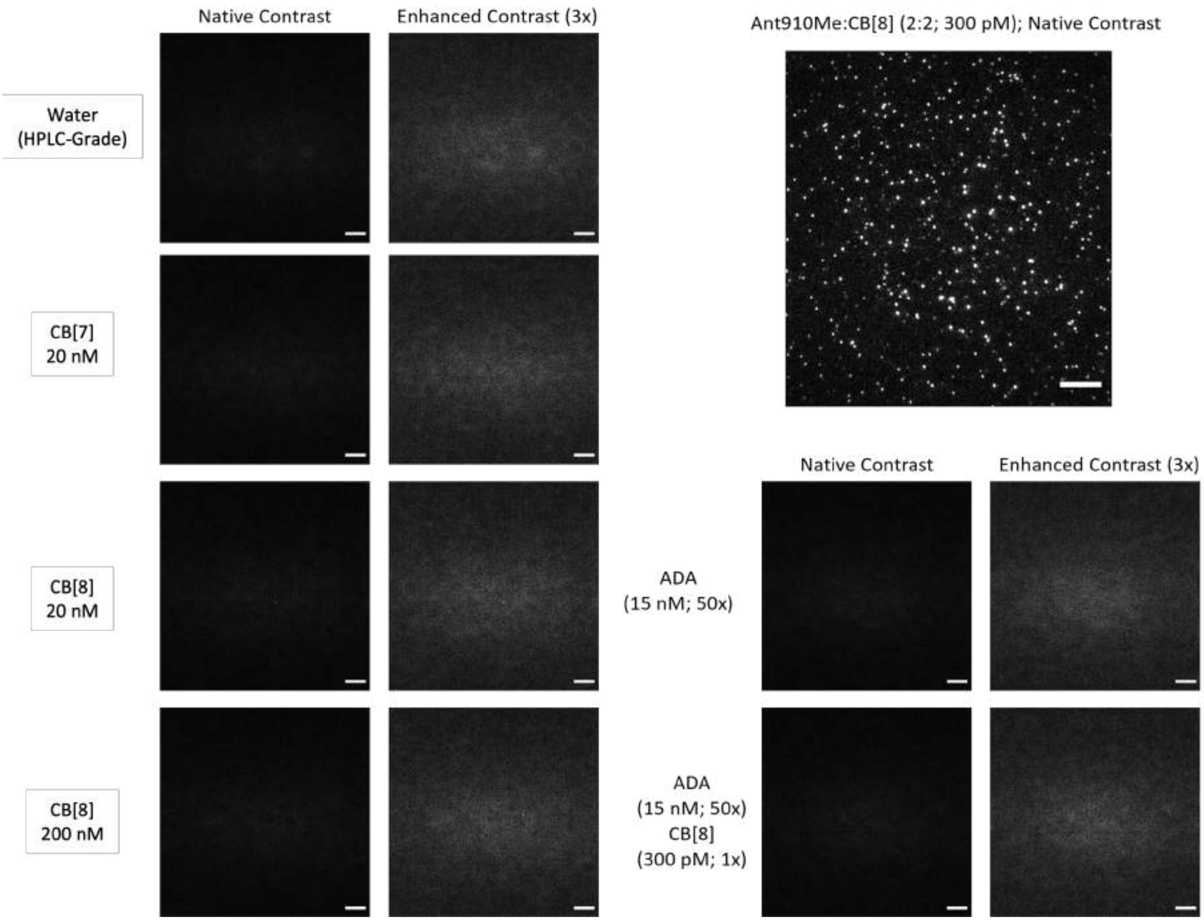

Figure S8: Single-molecule controls of water (HPLC-grade), CB[7] (20 nM), CB[8] (20 nM, 200 nM), adamantylamine (ADA; 15 nM; 50x excess), and ADA (15 nM) + CB[8] (300 pM). Native contrast and enhanced contrast (3x; contrast set to 1/3 of native contrast) are provided. Native contrasts show black backgrounds compared to single-molecule localizations obtained with Ant910Me samples (example localization provided for Ant910Me•CB[8] complex). Enhanced contrasts further reveal the lack of localizations across all control samples. For all images, the scale bar is 5 μm.

Table S2: Single molecule localizations detected for water (HPLC-grade), CB[7], and CB[8] controls. The lack of localizations for CB[7] and CB[8] controls reveal their utility for background-free single molecule microscopy.

Control Sample	Average Number of Localizations (n≥3)
Water	0
CB[7]; 20 nM	<1
CB[8]; 20 nM, 200 nM	1, 1.33
Adamantylamine; 15 nM (50 x excess)	0
Adamantylamine + CB[8]; 15 nM + 300 pM	2.75

2.5 Example trajectories of 2Ant910Me·2CB[8]

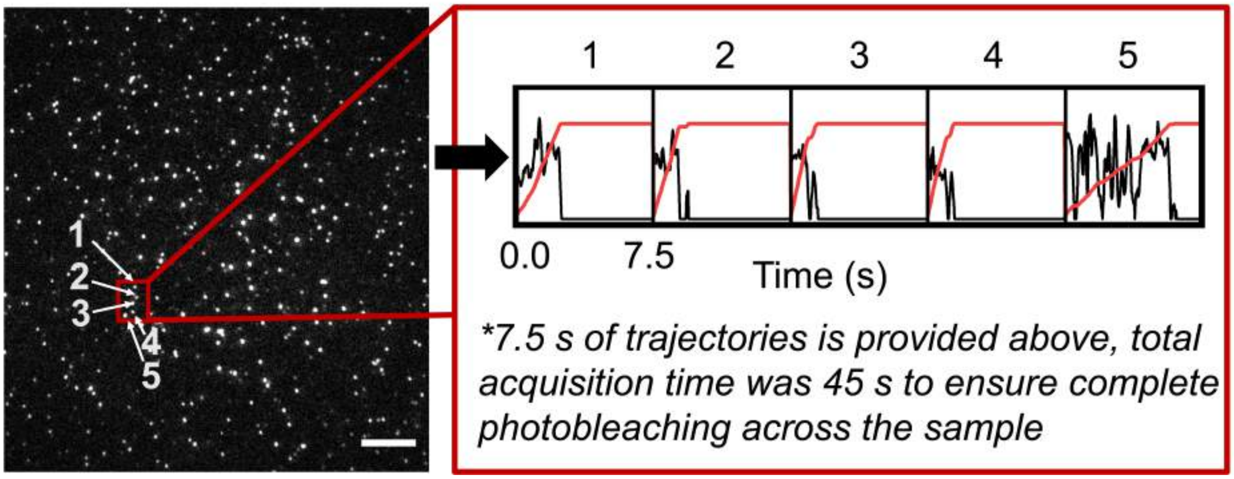

Figure S9: Examples of single-step photobleaches from 2Ant910Me•2CB[8] indicating that localizations correspond to single emitters, scale bar is 5 μm.

2.6 SMM of Ant910Me and CB[8] at various stoichiometric ratios

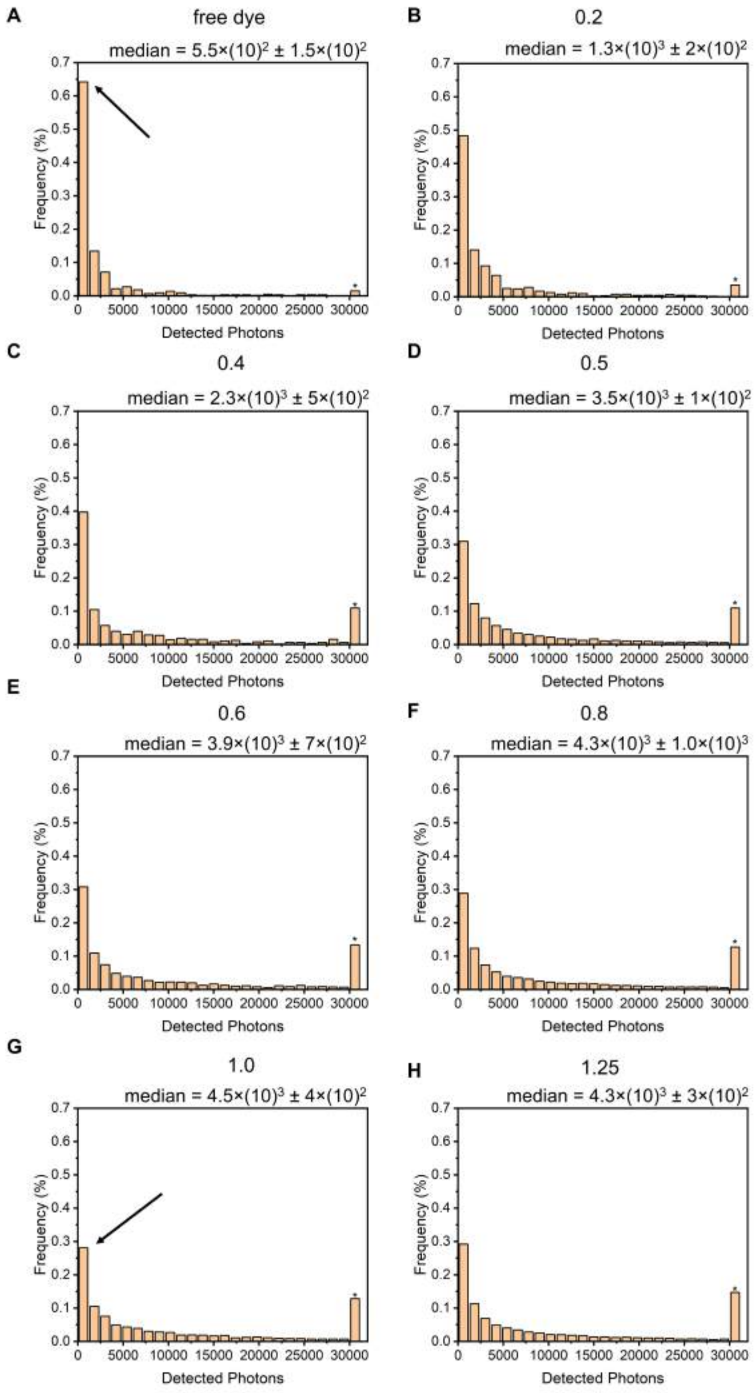

Figure S10: Single-molecule stoichiometry trials with varied ratios of Ant910Me to CB[8]; the median photons detected at each ratio is reported (y-axis), **A** free dye (0:1), **B** 0.2:1, **C** 0.4:1, **D** 0.5:1, **E** 0.6:1, **F** 0.8, **G** 1:1, **H** 1.25:1.

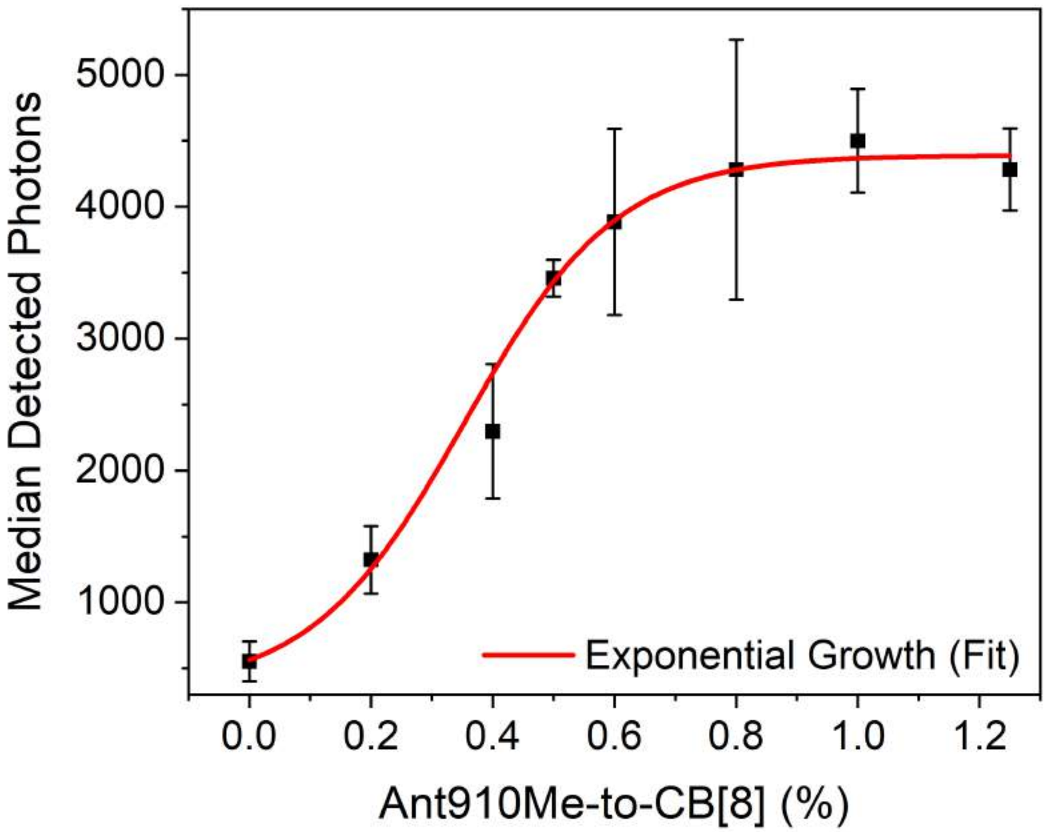

Figure S11: Overview of relationship between median detected photons and ratio of Ant910Me to CB[8]. Values are extracted from single-molecule experiments with varied ratios of Ant910Me and CB[8].

2.7 SMM of 2Ant910Me·2CB[8] complexes at various concentrations

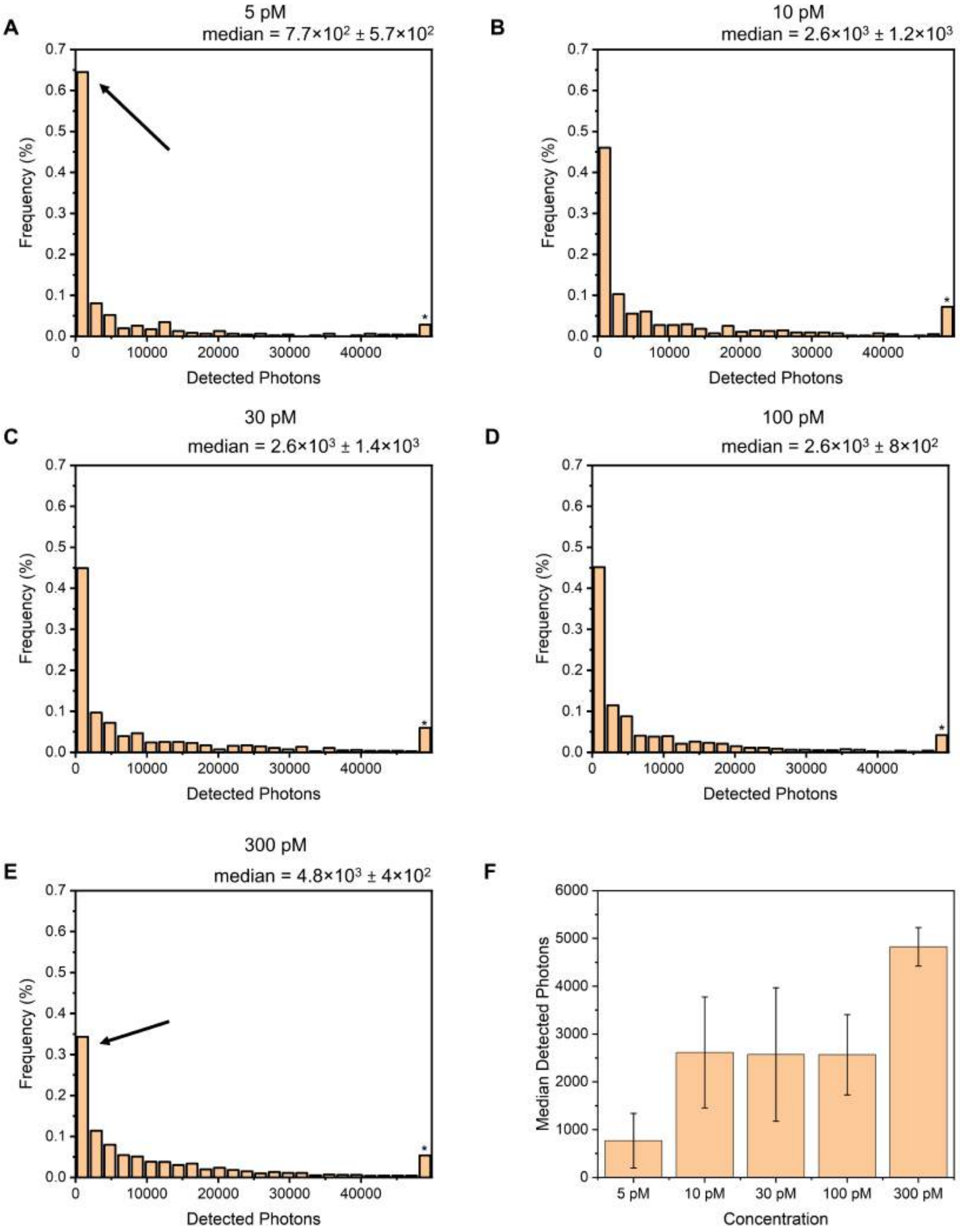

Figure S12: 2Ant910Me·2CB[8] single-molecule experiments at different concentrations, **A** 5 pM, **B** 10 pM, **C** 30 pM, **D** 100 pM, **E** 300 pM.

2.8 SMM Histogram Disjoints as a Function of Threshold (%)

The histogram disjoint is a measure of the non-overlap between two histograms. A value of 2 (200% representation) means that 100% of both histograms do not overlap. A value of 1 (100% representation) means that both histograms perfectly overlap. By scanning over a range of detection thresholds, we can find the optimal positions where two histograms non-overlap (producing the largest difference between the histograms). For the samples (Ant910Me *vs.* 2Ant910Me•2CB[8], Ant910Me•2CB[7] *vs.* 2Ant910Me•2CB[8], and Ant910Me•2CB[7] *vs.* 2Ant910Me•2CB[8] in agarose), this position is at a threshold of 82% (with 2% standard deviation). However, to better reduce the number of false positives in single fluorophore colorized images (as an 82% threshold means there is an 18% false positive rate), a 90% threshold was used (we note its position in the histogram disjoint is still close to the peak).

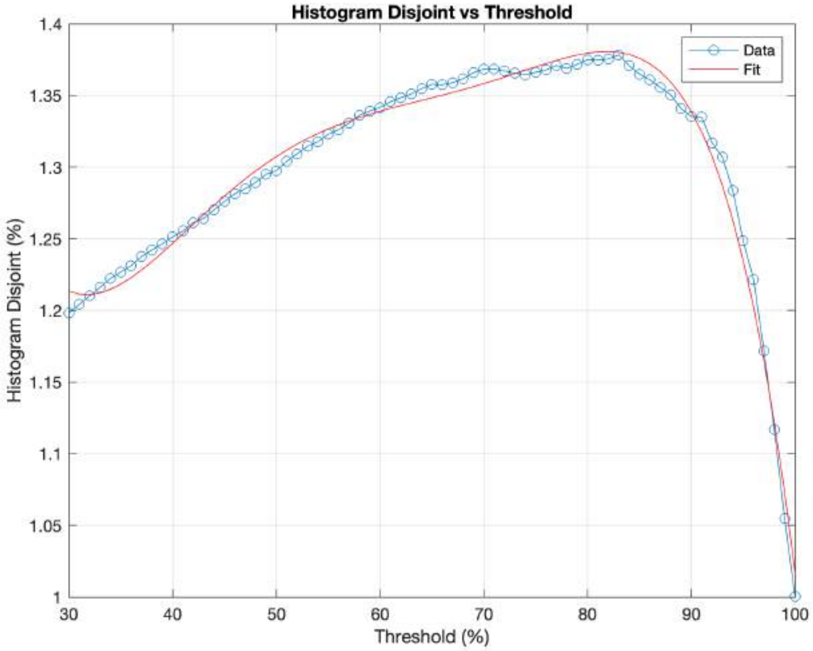

Figure S13: Histogram disjoint as a function of detection threshold for Ant910Me and 2Ant910Me•2CB[8] in solution. The red curve is the best-fit. A threshold value of 83% (per fitting) produces the greatest disjoint (least overlap between histograms).

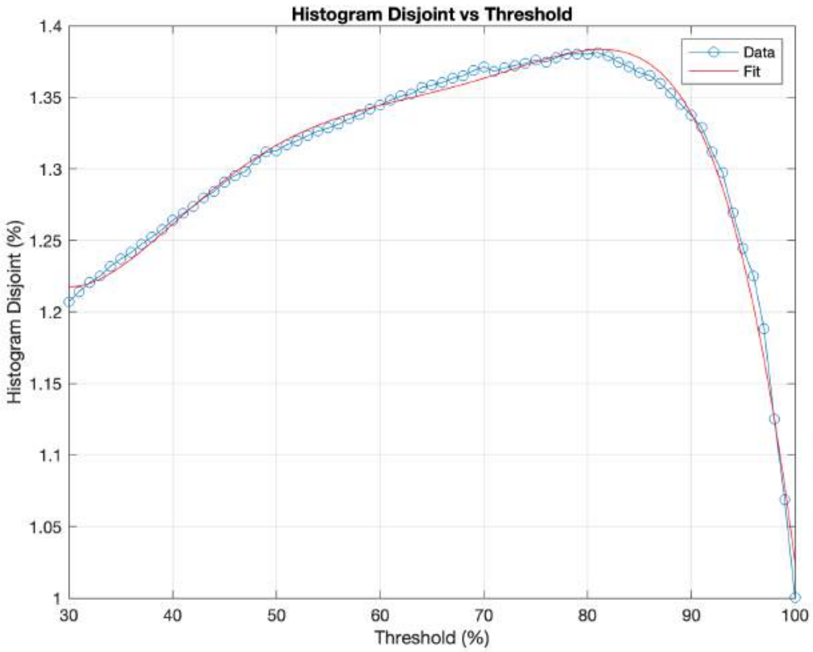

Figure S14: Histogram disjoint as a function of detection threshold for Ant910Me•2CB[7] and 2Ant910Me•2CB[8] *in solution*. The red curve is the best-fit. A threshold value of 83% (per fitting) produces the greatest disjoint (least overlap between histograms).

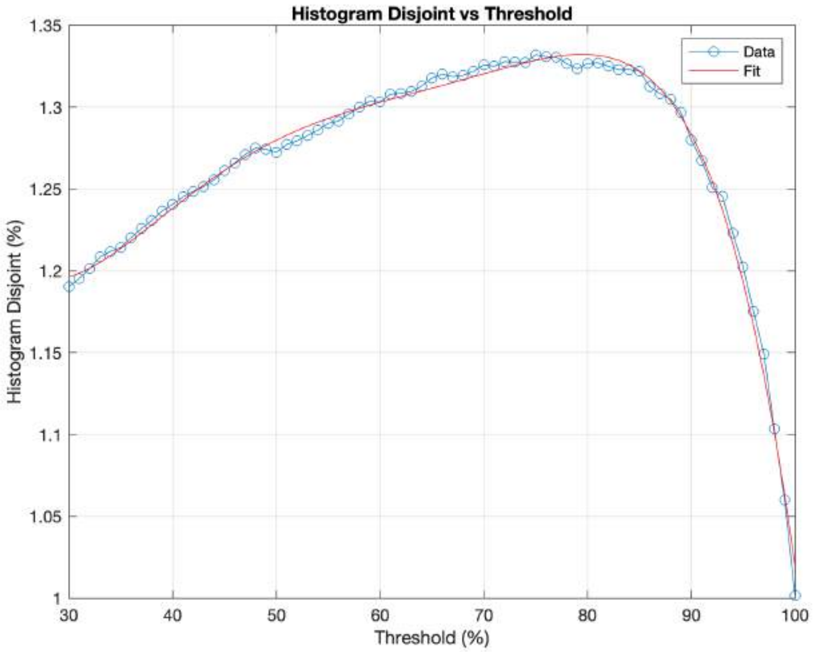

Figure S15: Histogram disjoint as a function of detection threshold for Ant910Me•2CB[7] and 2Ant910Me•2CB[8] *in agarose*. The red curve is the best-fit. A threshold value of 80% (per fitting) produces the greatest disjoint (least overlap between histograms).

2.9 SMM comparison of Ant910Me·2CB[7] and 2Ant910Me·2CB[8]

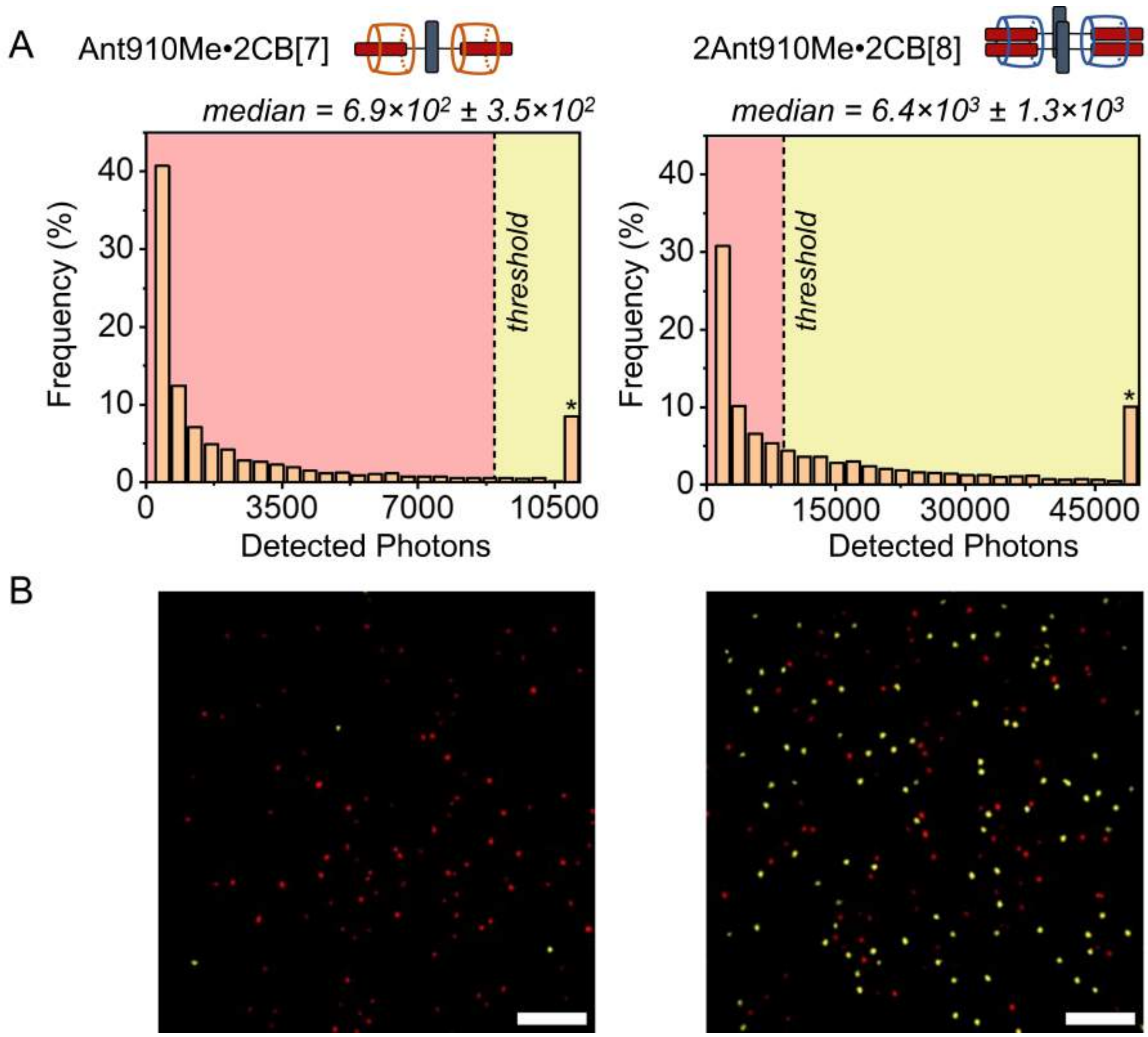

Figure S16: **A** Ant910Me·2CB[7] (left) and 2Ant910Me·2CB[8] (right) single-molecule histograms. The thresholded value (dashed line) was at 8958 detected photons (90th percentile of Ant910Me·2CB[7] histogram). **B** Colorized (red, yellow) localizations below and above threshold respectively for each sample, scale bar is 5 μm.

2.10 SMM Evaluation of Rho6G•CB[7]

Rhodamine 6G (Rho6G) has been shown to produce static single-molecule localizations when complexed with CB[7] to form a 1:1 complex, Rho6G•CB[7].[S10] To provide a comparison between the brightness of Rho6G and Ant910Me, we performed quantitative SMM on Rho6G•CB[7], Figure **S17**. We observe the single-molecule brightness of Rho6G•CB[7] to be slight elevated (1200 detected photons) as compared to free Ant910Me and Ant910Me•2CB[7] (700-800 detected photons). This can be rationalized as the quantum yield of Rho6G is near unity, whereas it is only 85% for Ant910Me•2CB[7].[S3,S4] The single-molecule brightness of Rho6G•CB[7], however, remains 5-6x less than that of the 2Ant910Me•2CB[8] complex. As Rho6G does not form a 2:2 complex with CB[8], we are unable to make a direct comparison in this case.

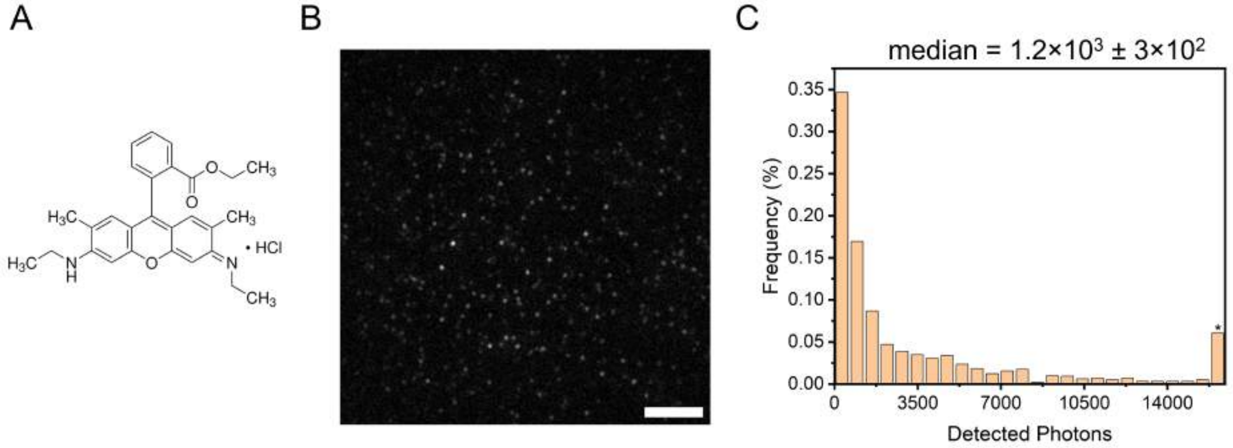

Figure S17: A Chemical structure of Rhodamine 6G **B** Single-molecule image from depositing Rho6G•CB[7], scale bar is 5 μm **C** Histogram of photons detected from Rho6G•CB[7].

2.11 Competitive displacement study

Figure **S18** shows the fluorescence titration of adamantylamine (ADA) into a 5 μM solution of 2Ant910Me•2CB[8]. The observed decrease in fluorescence in indicative of the displacement of Ant910Me from the CB[8] cavity. Although the fluorescence begins to decrease upon the addition of ADA, between 20-30 molar equivalents of ADA are required for the fluorescence to reach a stable baseline.

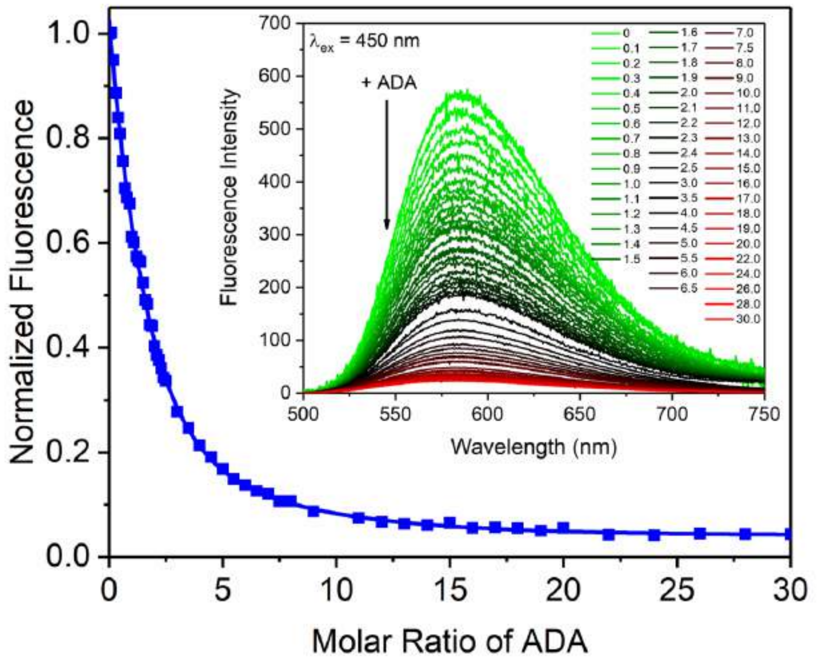

Figure S18: Fluorescence titration of adamantylamine (ADA) into 5 μM 2:2 Ant910Me•CB[8] complex under 450 nm excitation.

SMM images of the addition of ADA to 2Ant910Me•2CB[8] at various equivalents are shown in Figure **S19**. The median photons observed at each of the selected equivalents and the corresponding blue (free Ant910Me) : yellow (2Ant910Me•2CB[8]) values are described.

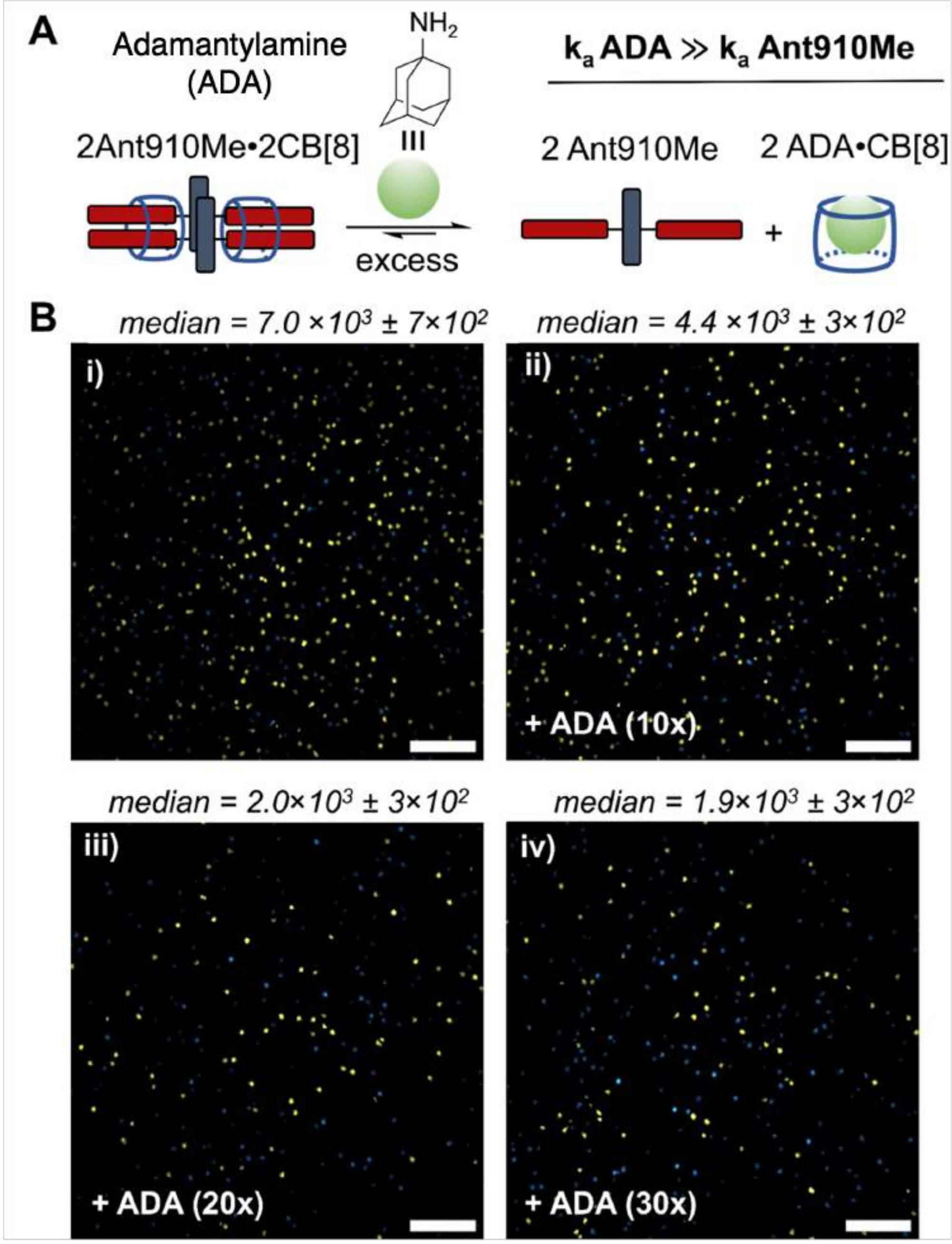

Figure S19: **A** Schematic overview of competitive displacement of **Ant910Me** from the CB[8] cavity by adamantylamine (ADA) **B** Colorized single-molecule localizations of 2Ant910Me•2CB[8] i.) before addition of ADA, ii-iv.) after the addition of ADA - ii.) 10 molar equivalents, iii.) 20 molar equivalents, iv.) 30 molar equivalents, scale bar is 5 μm. The ratio of blue-to-yellow for each sample is as follows: i.) 56%-44%, ii.) 63%-36%, iii.) 75%-25%, iv.) 80%-20%.

2.12 SMM comparison of free Ant910Me and 2Ant910Me•2CB[8] in Agarose

Ant910Me and 2Ant910Me•2CB[8] were loaded into separate agarose hydrogels (3 wt%) and imaged using SMM, Figure **S20**. Ant910Me shows a comparable number of median detected photons compared to in solution. Stoichiometric differentiation between free guest and 2Ant910Me•2CB[8] is achieved, analgous to solution measurements.

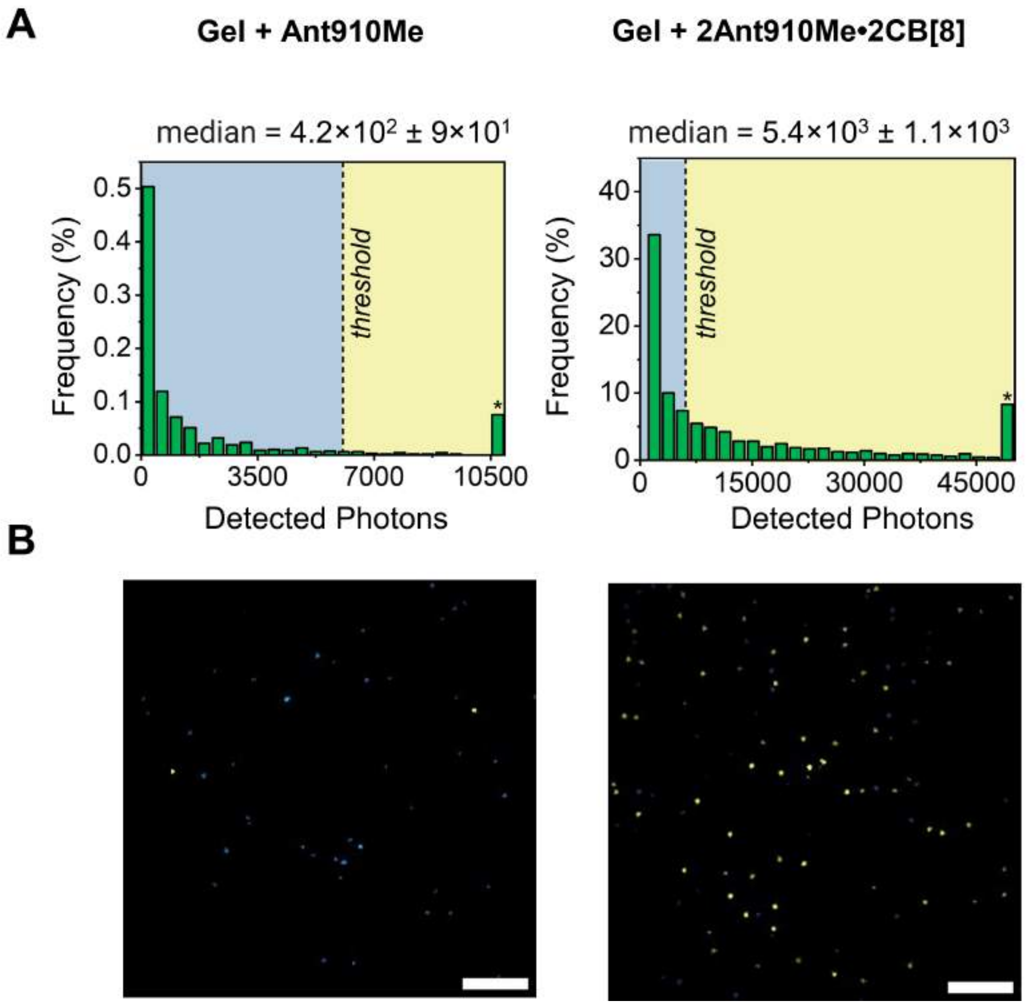

Figure S20: A Single-molecule histograms of detected photons for free Ant910Me (left) and 2Ant910Me•2CB[8] (right) in 3 wt% agarose gels. The thresholded value (dashed line) was set to the 90th percentile of Ant910Me in agarose histogram (at 6066 detected photons) **B** Colorized (blue, yellow) localizations below and above threshold respectively for each sample, scale bar is 5 μm.

2.13 Additional Single-Molecule Images

2.13.1 Single-Molecule Images for Figure 2

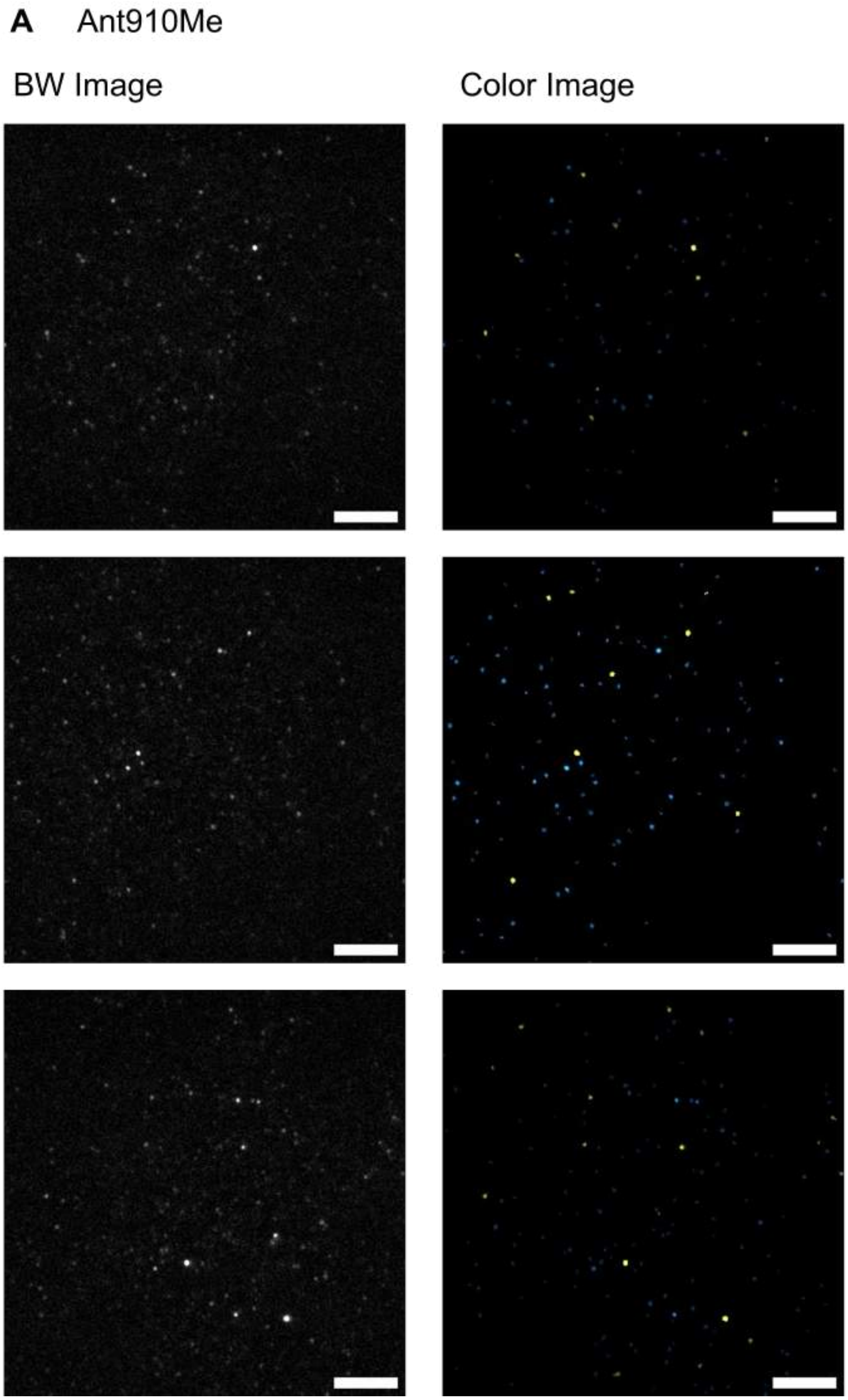

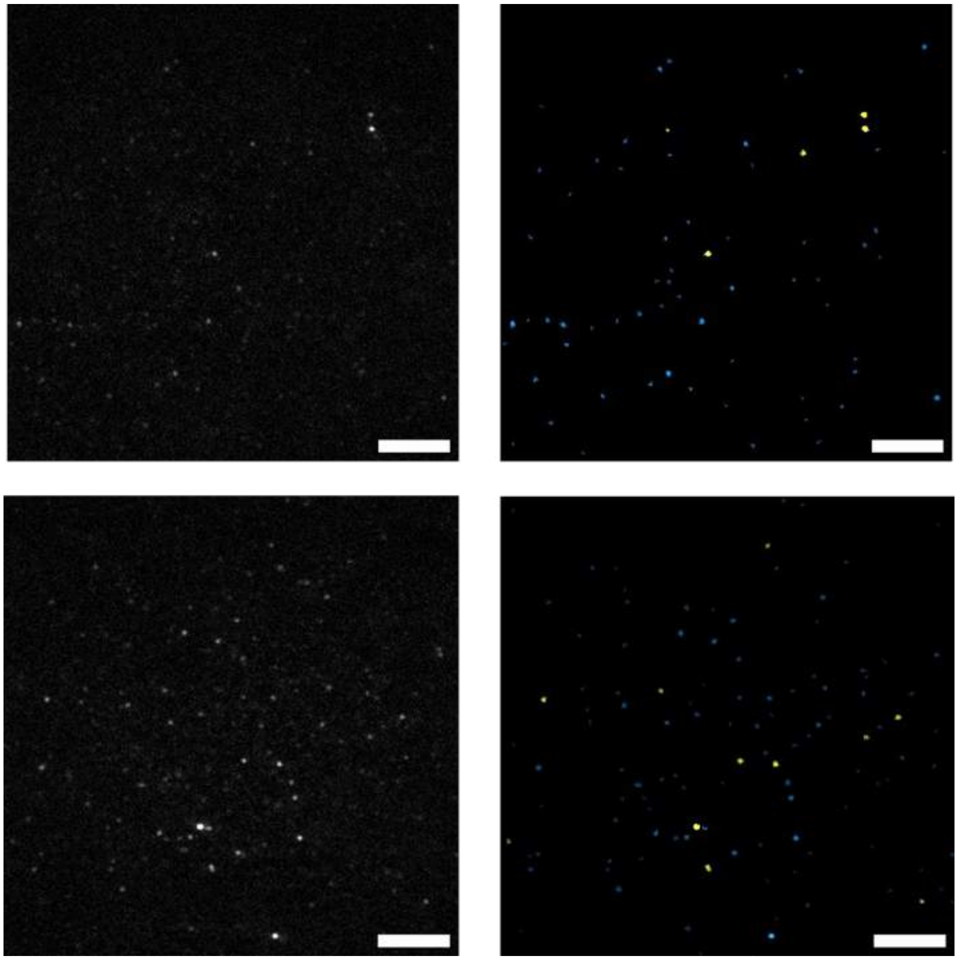

B 2Ant910Me•2CB[8]

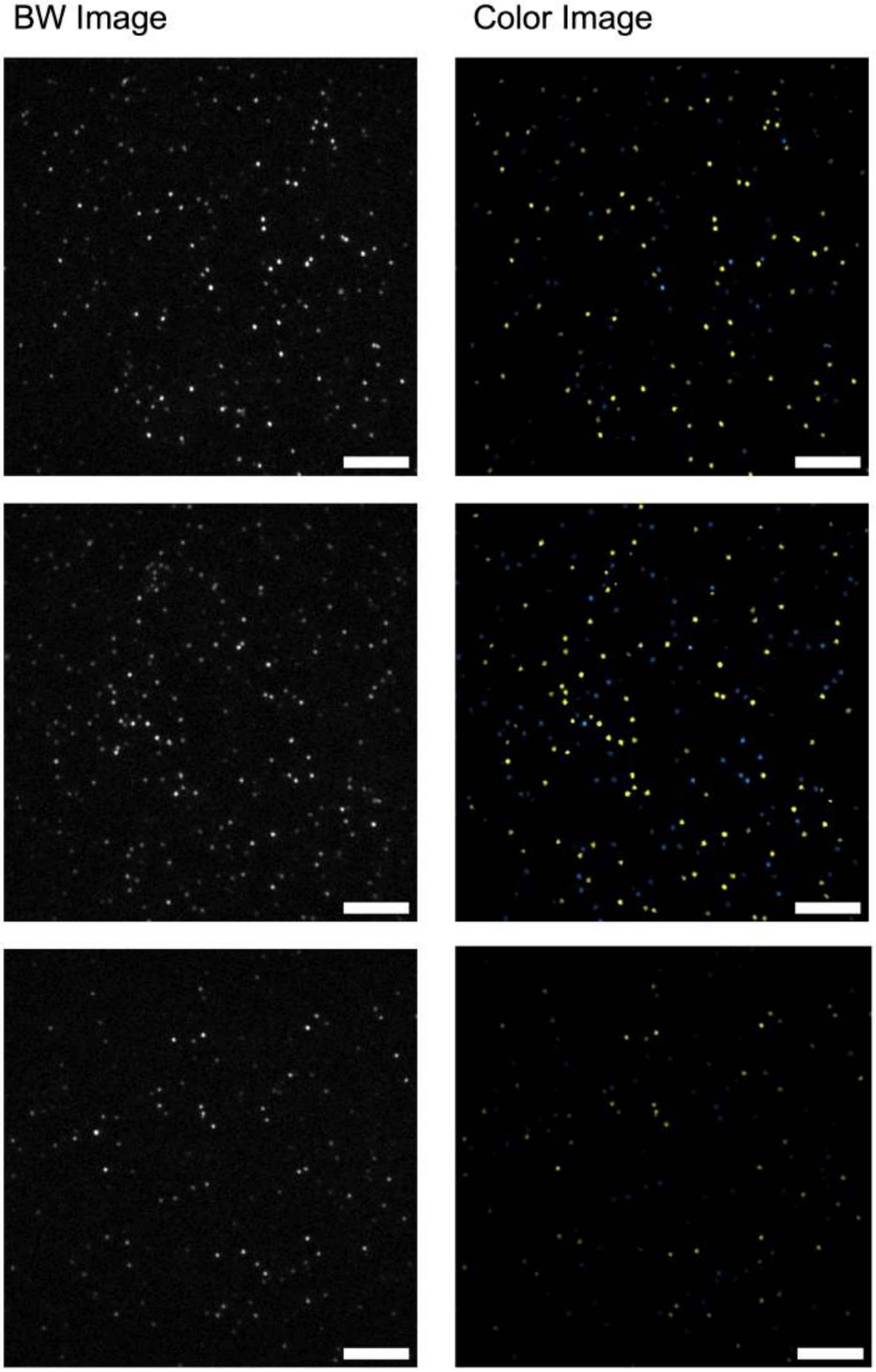

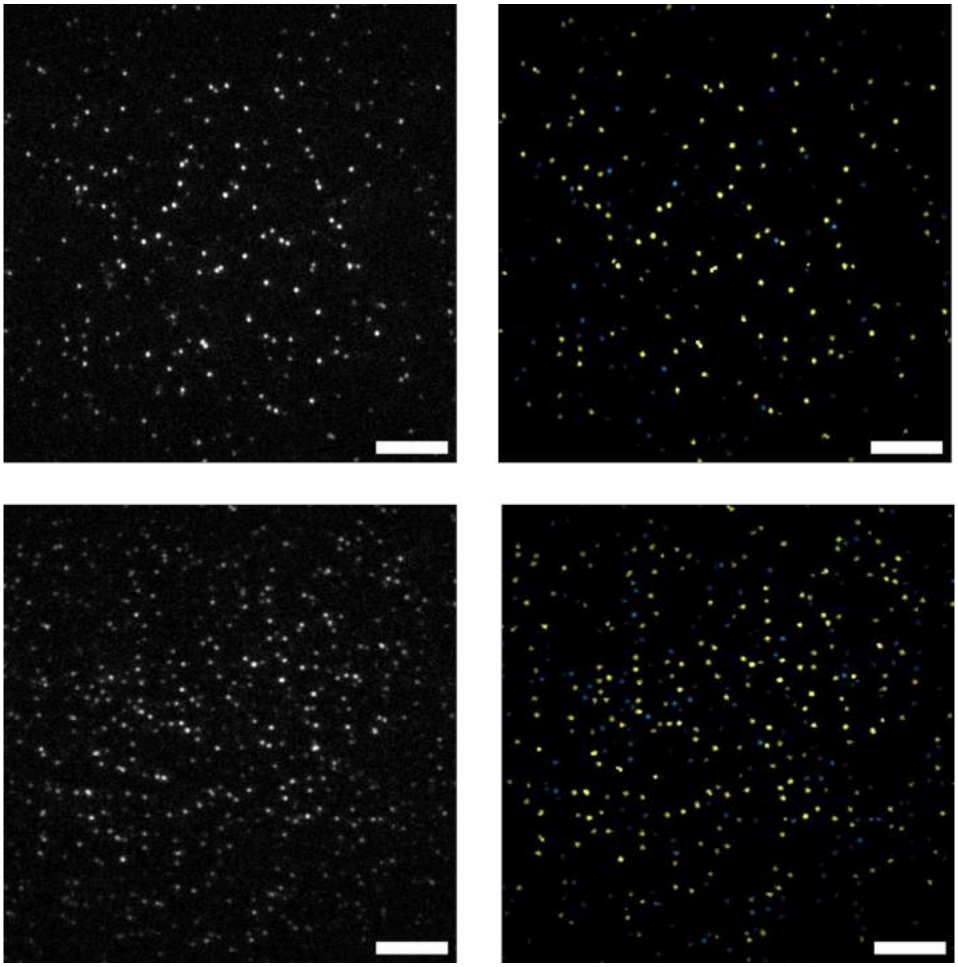

Figure S21: A. Single-molecule black and white (B&W) and colorized images for Ant910Me (free-dye) for 5 trials (encompassing 4 days of experiments) with the sample deposited at 1 nM. The first image can be found in Figure 2 (main text). **B.** Single-molecule black and white (B&W) and colorized images for 2Ant910Me•2CB[8] complex for 5 trials (encompassing 5 days of experiments) with the sample deposited at 300 pM. The first image can be found in Figure 2 (main text). For all images the scale bar is 5 μm.

2.13.2 Single-Molecule Images for Figure 3

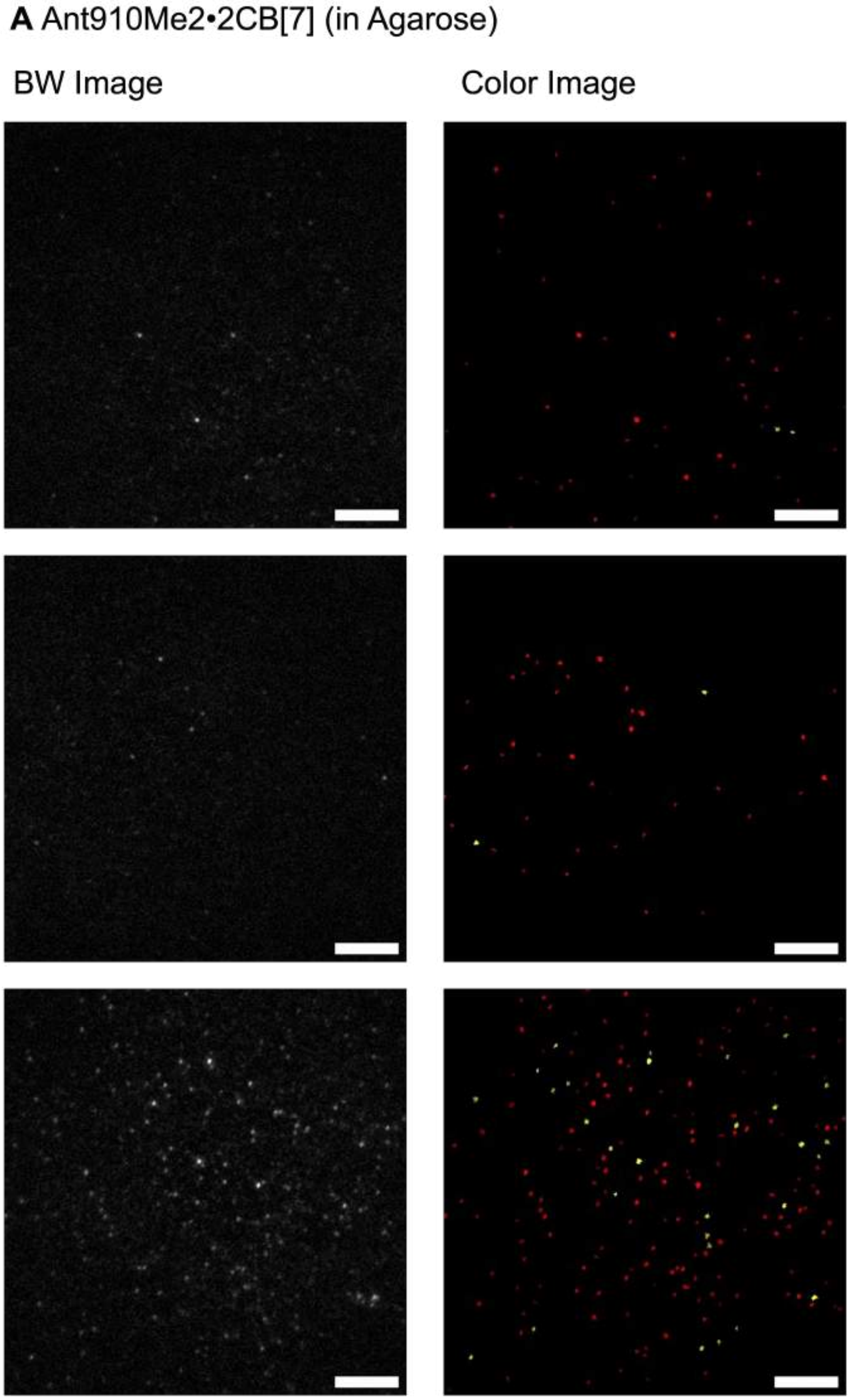

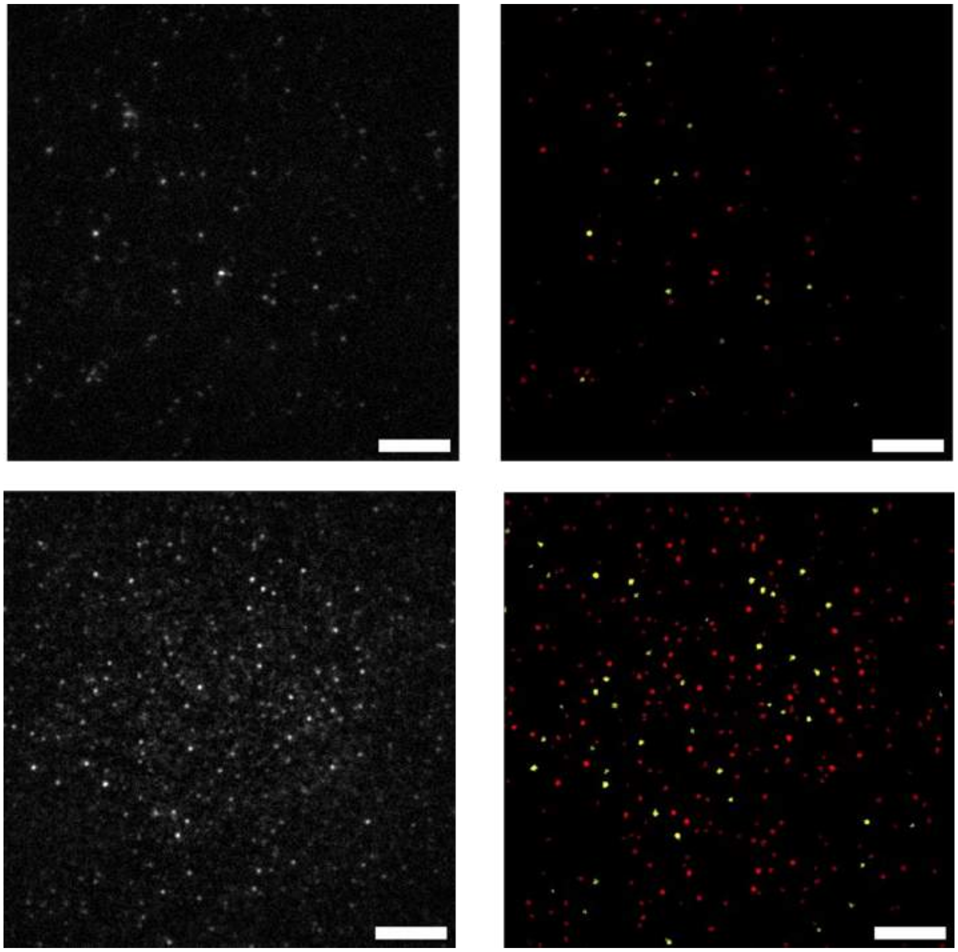

B 2Ant910Me•2CB[8] (in Agarose)

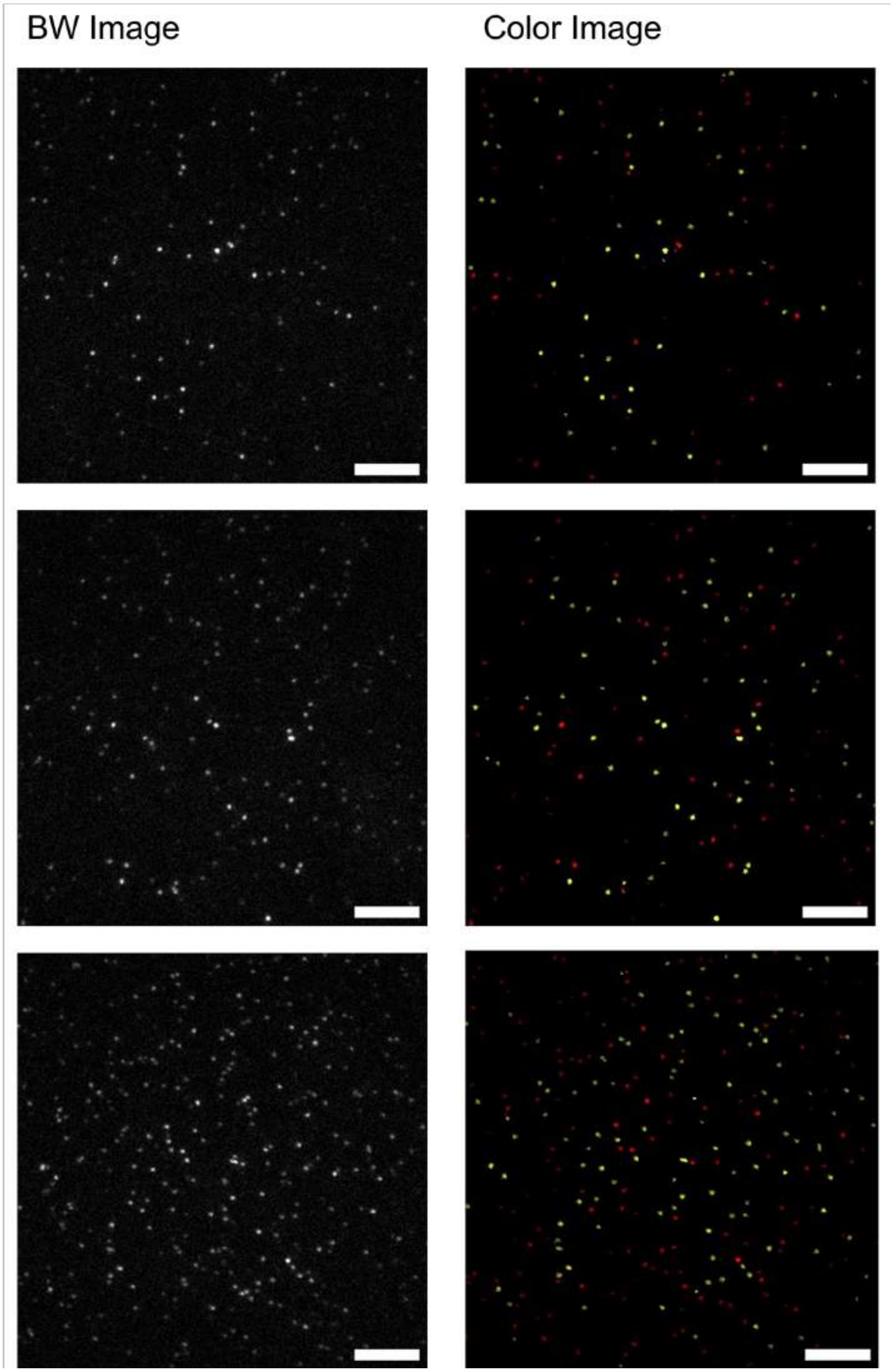

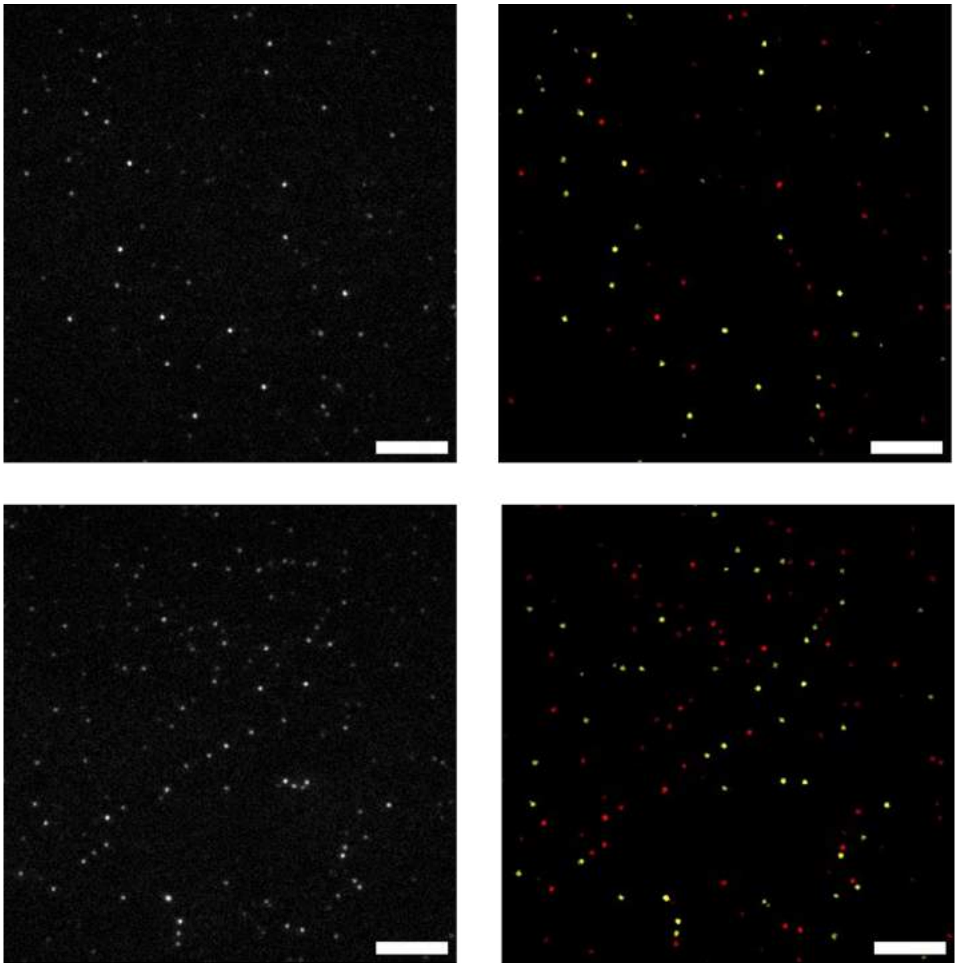

Figure S22: **A.** Single-molecule black and white (B&W) and colorized images for Ant910Me•2CB[7] for 5 trials (encompassing 3 days of experiments) with the sample injected into the hydrogel at 100 nM. The first image can be found in Figure 3 (main text). **B.** Single-molecule black and white (B&W) and colorized images for 2Ant910Me•2CB[8] complex for 5 trials (encompassing 3 days of experiments) with the sample injected into the hydrogel at 10 nM. The first image can be found in Figure 3 (main text). For all images the scale bar is 5 μm.

3 Appendix: Image Analysis Procedure

3.1 FIJI Macro Script

3.1.1 Intensity and Global Background Correction

The first part of the FIJI script corrects the intensity and global background of the image. The goal of the intensity correction is to convert the camera counts to photons. The first correction to be made is the camera bias (offset from 0) and gain (e- to ADU multiplier). The Prime BSI sCMOS camera has a bias of 100 $\pm$ 2.5 and a gain of 4.0 $\pm$ 0.3, as shown in Figure **S23**A. Due to the low standard deviation of the bias and gain, average values are used to convert the ADU reading to photons. We also corrected for the quantum efficiency of the photodetector (photons to e-), accounting for the efficiency at the relevant emission wavelength (reported by Photometrics, efficiencies are between 90-95 %) of each dye or complex. The script next corrects the global background, as shown in Figure **S23**B. For the correction, an averaged Gaussian blur correction is obtained (sigma = 50) using the back 7 % of frames in the timelapse. The backframes are used for the Gaussian blur correction, when complete photobleaching of the single-molecule localizations would be exhibited (only background signal).

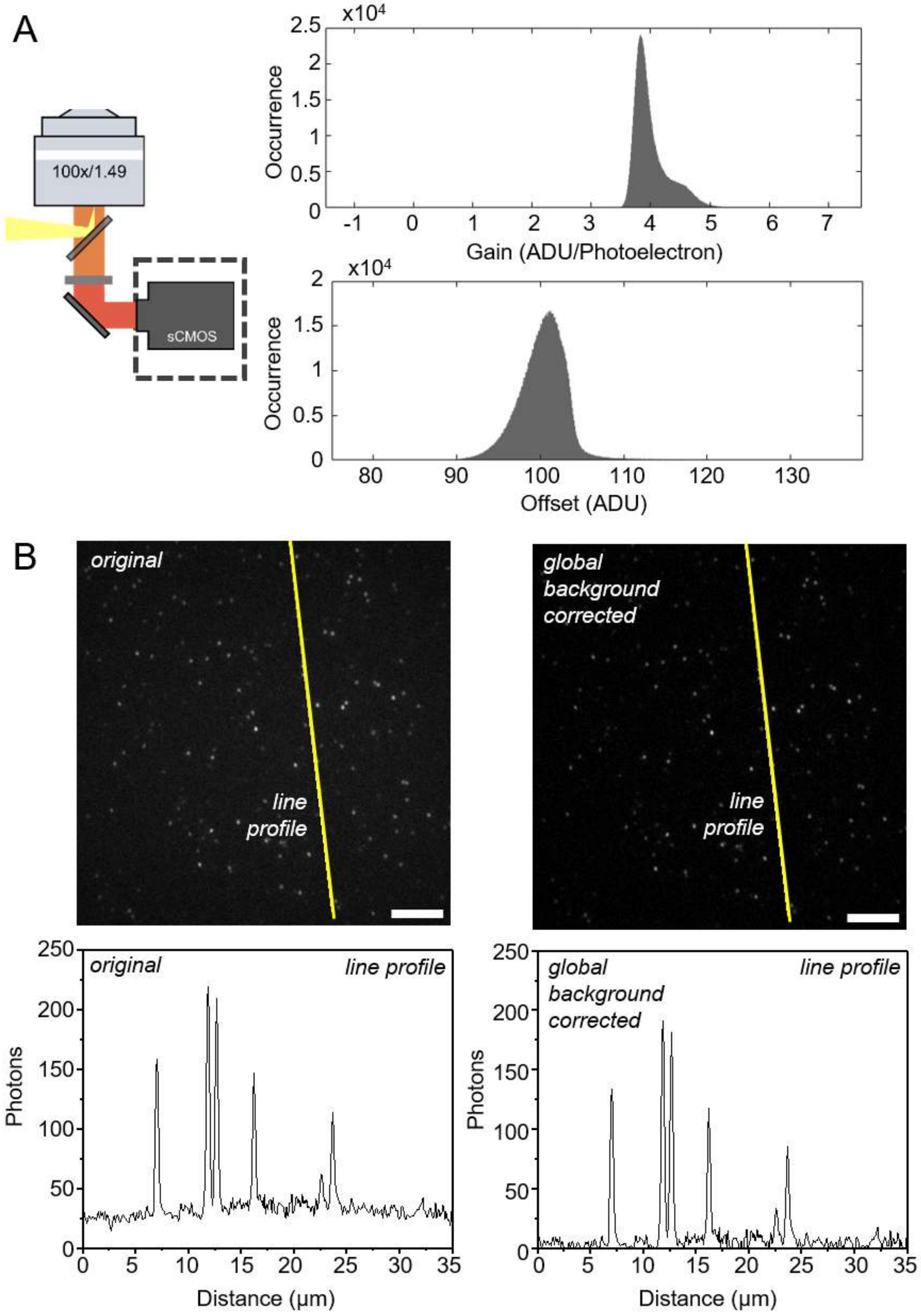

Figure S23: **A** sCMOS camera cartoon with bias (middle) and gain (right) reported. **B** (Top) Original image (bias and gain corrected but with no background subtraction) and global background corrected image, scale bar is 5 M. (Bottom) Example line profiles showing before and after global background correction.

3.1.2 Thresholding Prep: Noise Determination, Stack Processing

Localization selection (next section) is performed through a thresholding approach above the baseline noise, therefore the noise must first be determined. The baseline noise of the timelapse is determined by measuring the average signal of the averaged Gaussian blur (sigma = 50) of the back 7% of frames from the corrected timelapse. Like the global background correction, the backframes are used for determining the noise as nearly complete photobleaching of the single-molecule localizations is exhibited (only background signal remains). Before thresholding for identifying localizations, the stack is filtered/processed. First, the stacked is cropped; only the first 2 frames are retained for determining the 'on' trajectories. Cropping the stack is significant, as it selects for single molecules which are stuck on the glass (present immediately after laser excitation), as opposed to single molecules that are moving in-and-out of focus (appear randomly within the timelapse). The maximum pixel-by-pixel intensity of this timelapse is then projected for localization identification ('FrameMAX'), with an example shown in Figure **S24**A. Before analysis, the Frame-MAX is rolling ball corrected (sigma = 5) to further remove background for thresholding.

3.1.3 Localization Selection and Output

Identification of Single Molecules and Output. Identification of single molecules is performed through an iterative thresholding approach using the FrameMAX image. Using the 'Analyze Particles' macro in FIJI, signal multipliers above noise (3.0x, 4.0x, 5.0x, etc.) are scanned with bounding features applied (particle size in pixels and particle circularity; edge particles excluded), with an example shown in Figure **S24**B. The single-molecule boundaries identified by the analysis are therein expanded (2 pixel diameter, specified by the user) to encompass the full molecule. The thresholding protocol (single molecules identified at S/N, expansion, local background measurement) proceeds from low S/N to high S/N until no further particles are identified. Finally, the localizations (ROIs) identified by each threshold are overlaid. Since it is possible for the same localization to appear across multiple thresh-

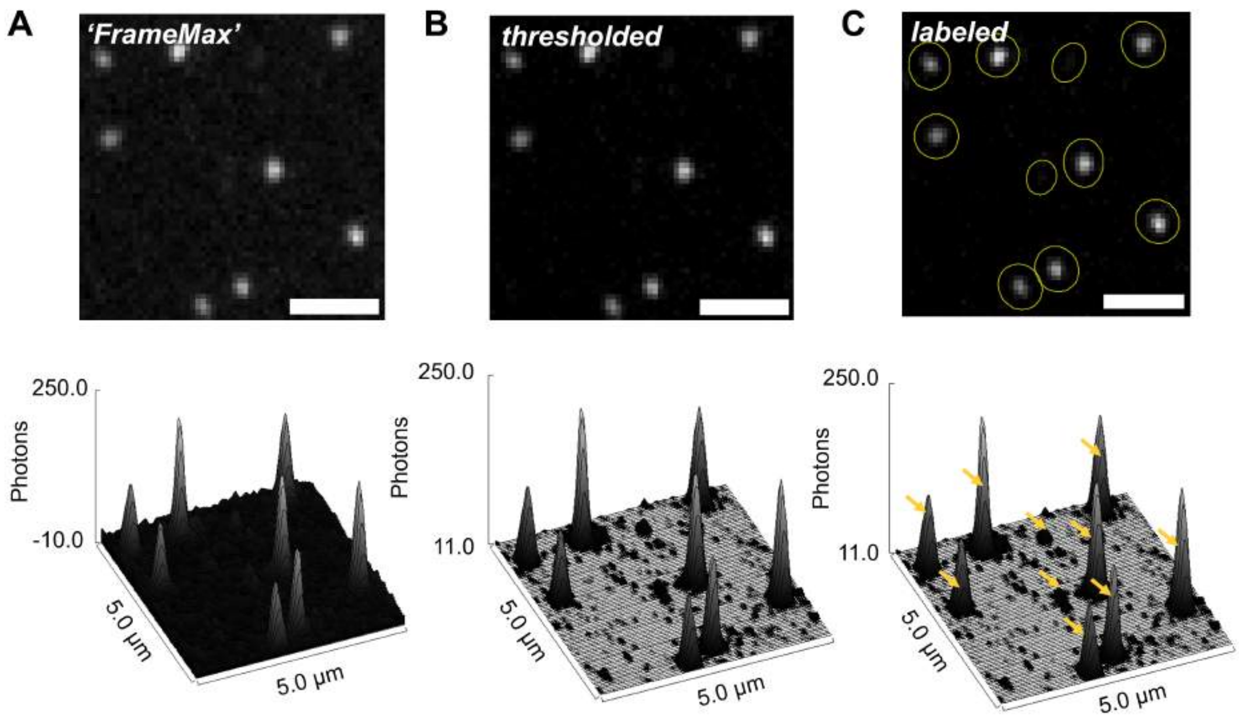

Figure S24: **A** 'FrameMax' localizations (scale bar is 1.5 M) and 3D surface plot before thresholding (average noise is 3.7 photons). **B** Thresholded localizations (3.0x) above noise (scale bar is 1.5 M) and thresholded 3D surface plot. **C** Labeled peaks and 3D surface plot (3.0x thresholded axis shown),scale bar is 1.5 M. Peaks are shown that achieve an on-state in MatLab 2D fitting (2 peaks were excluded).

olds, overlapping localizations between thresholds, which correspond to identical particles, are merged together. The final set of ROIs, corresponding to single molecular localizations scanned across all thresholds (an example is represented in Figure **S24**C, are therein used to export the single-molecule trajectories and local backgrounds for analysis.

3.2 Matlab Script

FIJI (ImageJ) is specialized for processing images, whereas processing numerical data is best done in a separate programming platform (Matlab, Python, etc.). We therefore wrote a Matlab script to process numerical localization ROI outputs from FIJI.

3.2.1 Script 1: 2D Gaussian Fitting and Determining Median Detected Photons

Using the ROIs exported from FIJI, each localization is processed to determine the photons detected. To do this, localization stacks are cropped from the single molecule stack. For each localization, fitting with a 2D Gaussian is performed (2 orthogonal 1D Gaussians centered at the sub-shot fit determined peak; Gaussian fits include a term for local background) frame-by-frame until the localization has turned off (localization must be off for 3 frames). Using the 2D fit parameters, the frame-by-frame volume of the 2D Gaussian is determined, which corresponds to the photons detected per frame. Summing across all frames, the total detected photons is thereby determined. The median detected photons across all trials for each molecule and the standard deviation (error) between trials is reported.

3.3 Getting Started: Beginner-Friendly Code Guide

For access to the codes, download the files at our laboratory Github.
Link: https://github.com/scherman-group/JACS-Single-molecule-stoichiometry-2024.git

Part 1: FIJI

Single-Molecule Brightness Code BrightnessV27 is a FIJI code that can identify single-molecules through loop-thresholding and outputs information about single-molecule ROIs, trajectories, etc. It can also calculate the brightness of single-molecule images through an integration approach (with automatically generated histogram output). However, we note this approach is not accurate when the single-molecule dyes are turned on only a small percent of the total number of frames, which is very common in single-molecule imaging. Instead, trajectories should be post-processed by FluorTrajAnal 2pt6 and 3pt1 codes, which perform 2D Gaussian fitting to identify 'on' trajectories (and filter out 'off' trajectories which add to noise). BrightnessV27 has a simple GUI and is designed to be used completely hands-off. It requires one package to be installed, 'BAR', if histogram plotting is enabled. Step-by-Step

Guide: 1. Open FIJI. 2. Open single-molecule stack. 3. Run plugin, Plugin->Macros->Run..., select code. 4. Follow prompts of code (modify as needed, default settings are typically a good starting place). 5. Make sure outputs are in an easy-to-find folder. Let the code execute.

ColorProcessing + LUTs ColorProcessingV4 is a FIJI code that can generate color-thresholded single-molecule images. It requires one package to be installed, 'BAR'. It should be run only after BrightnessV27 and the FluorTrajAnalysis workup, or with a code that generates comparable outputs. ColorProcessing requires several inputs.

First, it requires a single frame image of the single-molecule image in which to color. This is generated by BrightnessV27 under Folder Label 5 (5_frame_average_stacks_threshold).

ColorProcessing also requires a ROIs to threshold. This is generated by Brightness V27 under Folder Label 7 (7_ROIs). In the manuscript, Threshold_SUM (the sum from the entire threshold sweep), 01_ROIset_SUM (not expanded) was always used.

ColorProcessing lastly requires a CSV table of photon values. This is generated by FluorTrajAlternate2pt6 or 3pt1 (parallel version of code). Note: the first column of the CSV MUST say 'Budget'. With FluorTrajAlternate2pt6 or 3pt1 output, this column title will need to be entered in manually. ColorProcessing 2-color LUTs are provided. The LUT used is specified in the FIJI file (//Run Color Coder).

Part 2: Matlab

'FluorTrajAnalV2pt6.m/FluorTrajAnalV3pt1.m (V3 is for multiple single-molecule time-lapses)

FluorTrajAnal is a MatLab code that post-processes BrightnessV27 output to produce detected photon values. Version 2 can process one single-molecule timelapse/ROI file and is useful for testing, as it contains a few extra test options (for example checking single frames in the single-molecule timelapse). Version 3 can process multiple single-molecule

timelapses/ROI files without re-starting MatLab and is designed to be left running for long stretches of time.

For V2, the single-molecule timelapse location and ROI location must be specified (see code for instructions). For V3, the root directors for single-molecule timelapses and ROIs must be specified (see code for instructions). This means all single-molecule timelapses and ROIs must be in respective directories together.

Step-by-Step Guide:

1. Open MatLab.
2. Open either V2 or V3 code.
3. Specify folder locations for files needed to generate quantitative outputs.
4. Run MatLab code.

'FitFunctTest.m'

FitFunctTest.m is a fitting function that is required for the use of FluorTrajAnal scripts. This code must typically exist in the same folder as the FluorTrajAnal.m code.

Other MatLabCodes:

'Bootstrapper.m'

Bootstrapper (1 and 2) are MatLab codes that can be run to determine histogram spread (these could be run on compiled photon budget outputs). Bootstrapper 1 is the traditional bootstrapper algorithm (see Chernick, M. R. Resampling Methods. WIREs Data. Mining. Knowl. Discov. 2012, 2, 255-262), whereas Bootstrapper 2 is a variant. However, we found that standard deviation between trials is much larger than bootstrapper error within a trial, therefore standard deviation is reported in the manuscript and the bootstrapper code did not end up being needed.

'PercentLoop.m'

Loops over percent threshold values to find a value of the overlap between two histograms (getting a value of 2 or 200% means there is no overlap between samples, 1 or 100% means

there is complete overlap between samples). Plots the overlap vs. the percent threshold for all values in the loop.

‘Photon Budget Compiler.m’

Photon budget compiler is a MatLab code that allows the merging of different detected photon trials into a single file. A cutoff for the maximum photons detected can be applied. The parallel version can merge detected photons not just within one folder (typically corresponding to a single date of experiments with n number of trials), but also merges multiple folders (corresponding to multiple dates of experiments). To make handling the compiled data easier, the parallel version creates folders for photons detected ‘budgets’ and median values ‘medians’ (used to determine standard deviation) with values from each folder.

‘Recover Gauss.m’

Recover Gauss is a MatLab code that can recover raw output from FluorTrajAnalV2/V3 very quickly (within seconds). Since the output of from these codes is exported in csv format, Recover Gauss re-imports these tables back into MatLab. ‘Recover Gauss’ can save the user a very large amount of time in comparison to re-running FluorTrajAnal codes again if small modifications to mathematical operations need to be performed.

References

(S1) Kim, J.; Jung, I.-S.; Kim, S.-Y.; Lee, E.; Kang, J.-K.; Sakamoto, S.; Yamaguchi, K.; Kim, K. New Cucurbituril Homologues: Syntheses, Isolation, Characterization, and X-ray Crystal Structures of Cucurbit[n]uril (n = 5, 7, and 8). *J. Am. Chem. Soc.* **2000**, *122*, 540–541.

(S2) Day, A.; Arnold, A. P.; Blanch, R. J.; Snushall, B. Controlling Factors in the Synthesis of Cucurbituril and Its Homologues. *J. Org. Chem.* **2001**, *66*, 8094–8100.

(S3) Wu, G.; Szabó, I.; Rosta, E.; Scherman, O. A. Cucurbit[8]uril-mediated pseudo[2,3]rotaxanes. *Chem. Commun.* **2019**, *55*, 13227–13230.

(S4) Wu, G.; Bae, Y. J.; Olesińska, M.; Antón-García, D.; Szabó, I.; Rosta, E.; Wasielewski, M. R.; Scherman, O. A. Controlling the Structure and Photophysics of Fluorophore Dimers Using Multiple Cucurbit[8]Uril Clampings. *Chem. Sci.* **2020**, *11 (3)*, 812–825.

(S5) Wu, G.; Huang, Z.; Scherman, O. A. Quantitative supramolecular heterodimerization for efficient energy transfer. *Angew. Chem. Int. Ed.* **2020**, *59*, 15963–15967.

(S6) Sungkaworn, T.; Rieken, F.; Lohse, M. J.; Calebiro, D. High-resolution Spatiotemporal Analysis of Receptor Dynamics by Single-molecule Fluorescence Microscopy. *J. Vis. Exp.* **2014**, *89*, e51784.

(S7) Jain, A.; Liu, R.; Ramani, B.; Arauz, E.; Ishitsuka, Y.; Ragunathan, K.; Park, J.; Chen, J.; Xiang, Y. K.; Ha, T. Probing cellular protein complexes using single-molecule pull-down. *Nature* **2011**, *473*, 484–488.

(S8) Mukherjee, S.; Thomas, C.; Wilson, R.; Simmerman, E.; Hung, S.-T.; Jimenez, R. Characterizing dark state kinetics and single molecule fluorescence of FusionRed and FusionRed-MQ at low irradiances. *Phys. Chem. Chem. Phys.* **2022**, *24*, 14310.

(S9) Fricke, F.; Beaudouin, J.; Eils, R.; Heilemann, M. One, two or three? Probing the stoichiometry of membrane proteins by single-molecule localization microscopy. *Sci. Rep.* **2015**, *5*, 14072.

(S10) Martyn, T. A.; Moore, J. L.; Halterman, R. L.; Yip, W. T. Cucurbit[7]uril Induces Superior Probe Performance for Single-Molecule Detection. *J. Am. Chem. Soc.* **2007**, *129 (34)*, 10338–10339.

(S11) Trani, J. M. D.; Cesco, S. D.; O'Leary, R.; Plescia, J.; do Nascimento, C. J.; Moitessier, N.; Mittermaier, A. K. Rapid measurement of inhibitor binding kinetics by isothermal titration calorimetry. *Nat. Commun.* **2018**, *9 (1)*, 1–7.

(S12) Moitessier, J. M. D. T. N.; Mittermaier, A. K. Complete Kinetic Characterization of Enzyme Inhibition in a Single Isothermal Titration Calorimetric Experiment. *Anal. Chem.* **2018**, *90 (14)*, 8430–8435.

(S13) Havel, V.; Yawer, M. A.; Sindelar, V. Real-time analysis of multiple anion mixtures in aqueous media using a single receptor. *Chem. Comm.* **2015**, *15*, 4666–4669.

(S14) Jahnke, N.; Krylova, O. O.; Hoomann, T.; Vargas, C.; Fiedler, S.; Pohl, P.; Keller, S. Real-Time Monitoring of Membrane-Protein Reconstitution by Isothermal Titration Calorimetry. *Anal. Chem.* **2014**, *86 (1)*, 920–927.

(S15) Lv, M.; Zhang, Y.-J.; Zhou, F.; Ge, Y.; Zhao, M.-H.; Liu, Y.; Yang, K.-W. Real-time monitoring of D-Ala-D-Ala dipeptidase activity of VanX in living bacteria by isothermal titration calorimetry. *Anal. Biochem.* **2019**, *578*, 20–35.

(S16) Zhang, Y.-J.; Wang, W.-M.; Oelschlaeger, P.; Chen, C.; Lei, J.-E.; Lv, M.; Yang, K.-W. Real-Time Monitoring of NDM-1 Activity in Live Bacterial Cells by Isothermal Titration Calorimetry: A New Approach To Measure Inhibition of Antibiotic-Resistant Bacteria. *ACS Infect. Dis.* **2018**, *4*, 16711678.

(S17) Liu, J.; Wang, C.; Jiang, Y.; Hu, Y.; Li, J.; Yang, S.; Li, Y.; Yang, R.; Tan, W.; Huang, C. Z. Graphene Signal Amplification for Sensitive and Real-Time Fluorescence Anisotropy Detection of Small Molecules. *Chem. Comm.* **2013**, *85 (3)*, 1424–1430.

(S18) Florea, M.; Kudithipudi, S.; Rei, A.; González-Álvarez, M. J.; Jeltsch, A.; Nau, W. M. A Fluorescence-Based Supramolecular Tandem Assay for Monitoring Lysine Methyltransferase Activity in Homogeneous Solution. *Eur. J. Chem.* **2012**, *18*, 3521–3528.

(S19) Ghale, G.; Lanctôt, A. G.; Kreissl, H. T.; Jacob, M. H.; Weingart, H.; Winterhalter, M.; M., W. Chemosensing Ensembles for Monitoring Biomembrane Transport in Real Time. *Angew. Chem. Int. Ed.* **2014**, *53*, 2762 –2765.

(S20) Tang, Y.; Achyuthan, K. E.; Whitten, D. G. Label-free and Real-Time Sequence Specific DNA Detection Based on Supramolecular Self-assembly. *Langmuir* **2010**, *26 (9)*, 6832–6837.

(S21) Florea, M.; Nau, W. M. Strong Binding of Hydrocarbons to Cucurbituril Probed by Fluorescent Dye Displacement: A Supramolecular Gas-Sensing Ensemble. *Angew. Chem.* **2011**, *123*, 9510–9514.

(S22) Zheng, Z.; Ren, S.; Geng, W.-C.; Cui, X.; Wu, B.; Wang, H. Monitoring Methionine Decarboxylase by a Supramolecular Tandem Assay. *Chem Asian J.* **2022**, *17*, e202200106.

(S23) Wang, K.; Cui, J.-H.; Xing, S.-Y.; Dou, H.-X. A calixpyridinium-based supramolecular tandem assay for alkaline phosphatase and its application to ATP hydrolysis reaction. *Org. Biomol. Chem.* **2016**, *14*, 2684–2690.

(S24) Barba-Bon, A.; Pan, Y.-C.; Biedermann, F.; Guo, D.-S.; Nau, W. M.; Hennig, A. Fluorescence Monitoring of Peptide Transport Pathways into Large and Giant Vesicles by Supramolecular Host–Dye Reporter Pairs. *J. Am. Chem. Soc.* **2019**, *141 (51)*, 20137–20145.

(S25) Prabodh, A.; Wang, Y.; Sinn, S.; Albertini, P.; Spies, C.; Spuling, E.; Yang, L.-P.; Jiang, W.; Bräse, S.; Biedermann, F. Fluorescence detected circular dichroism (FDCD) for supramolecular host–guest complexes. *Chem. Sci.* **2021**, *12*, 9420–9431.

(S26) Huang, C.-B.; Xu, L.; Zhu, J.-L.; Wang, Y.-X.; Sun, B.; Li, X.; Yang, H.-B. Real-Time Monitoring the Dynamics of Coordination-Driven Self-Assembly by Fluorescence-Resonance Energy Transfer. *J. Am. Chem. Soc.* **2017**, *139*, 94599462.

(S27) Tang, C.; Qian, Z.; Qian, Y.; Huang, Y.; Zhao, M.; Ao, H.; Feng, H. A fluorometric and real-time assay for -glucosidase activity through supramolecular self-assembly and its application for inhibitor screening. *Sens. Actuators B Chem.* **2017**, *245*, 282–289.

(S28) Liu, Z.; Zhou, W.; Li, J.; Zhang, H.; Dai, X.; Liu, Y.; Liu, Y. High-efficiency dynamic sensing of biothiols in cancer cells with a fluorescent -cyclodextrin supramolecular assembly. *Chem. Sci.* **2020**, *11*, 4791–4800.

(S29) Wolfe, A. J.; Hsueh, Y.-C.; Blanden, A. R.; Mohammad, M. M.; Pham, B.; Thakur, A. K.; Loh, S. N.; Chen, M.; Movileanu, L. Interrogating Detergent Desolvation of Nanopore-Forming Proteins by Fluorescence Polarization Spectroscopy. *Anal. Chem.* **2017**, *89 (15)*, 8013–8020.

(S30) Yang, M.; Yang, K.; Gao, B.; Wang, P.; Li, T.; Zheng, Y.; Pei, Y.; Pei, Z.; Lv, Y. A supramolecular nano-delivery system based on AIE PARP inhibitor prodrug and glycosylated pillar[5]arene for drug-resistance therapy. *Chem. Comm.* **2022**, *58*, 11147–11150.

(S31) Almeida, M. P.; Kock, F. V.; de Jesus, H. C.; Carlos, R. M.; Venâncio, T. Probing the acetylcholinesterase inhibitory activity of a novel Ru(II) polypyridyl complex and the supramolecular interaction by (STD)-NMR. *J. Inorg. Biochem.* **2021**, *224*, 111560.

(S32) Wenter, P.; Fürtig, B.; Hainard, A.; Schwalbe, H.; Pitsch, S. Kinetic investigation of

photoinduced RNA refolding by real-time NMR spectroscopy. *Angew. Chem. Int. Ed* **2005**, *44*, 2600–2603.

(S33) Rennella, E.; Sekhar, A.; Kay, L. E. Self-Assembly of Human Profilin-1 Detected by Carr–Purcell–Meiboom–Gill Nuclear Magnetic Resonance (CPMG NMR) Spectroscopy. *Biochemistry* **2017**, *56 (5)*, 692–703.

(S34) Rennella, E.; Cutuil, T.; Schanda, P.; Ayala, I.; Forge, V.; Brutscher, B. Real-Time NMR Characterization of Structure and Dynamics in a Transiently Populated Protein Folding Intermediate. *J. Am. Chem. Soc.* **2012**, *134 (19)*, 8066–8069.

(S35) Schanda, P.; Brutscher, B. Very Fast Two-Dimensional NMR Spectroscopy for Real-Time Investigation of Dynamic Events in Proteins on the Time Scale of Seconds. *J. Am. Chem. Soc.* **2005**, *127 (22)*, 8014–8015.

(S36) Schanda, P.; Forge, V.; Brutscher, B. Protein folding and unfolding studied at atomic resolution by fast two-dimensional NMR spectroscopy. *Proc. Natl. Acad. Sci. U.S.A.* **2007**, *104 (27)*, 11257–11262.

(S37) Fernandes, H.; Filgueiras, J. G.; de Azevedo, E. R.; Lima-Neto, B. S. Real time monitoring by time-domain NMR of ring opening metathesis copolymerization of norbornene-based red palm olein monomer with norbornene. *Eur. Polym. J.* **2020**, *140*, 110048.

(S38) Corra, S.; Bakić, M. T.; Groppi, J.; Baroncini, M.; Silvi, S.; Penocchio, E.; Esposito, M.; Credi, A. Kinetic and energetic insights into the dissipative non-equilibrium operation of an autonomous light-powered supramolecular pump. *Nat. Nanotechnol.* **2022**, *17*, 746–751.

(S39) Kang, J.; Lhee, S.; Lee, J. K.; Zare, R. N.; Nam, H. Restricted intramolecular rotation of fuorescent molecular rotors at the periphery of aqueous microdroplets in oil. *Sci. Rep.* **2020**, *10*, 16859.
